# Comprehensive multi-omics analysis of breast cancer reveals distinct long-term prognostic subtypes

**DOI:** 10.1038/s41389-024-00521-6

**Published:** 2024-06-13

**Authors:** Abhibhav Sharma, Julia Debik, Bjørn Naume, Hege Oma Ohnstad, Oslo Breast Cancer Consortium (OSBREAC), Kristine Kleivi Sahlber, Elin Borgen, Anne-Lise Børresen-Dale, Olav Engebråten, Britt Fritzman, Øystein Garred, Jürgen Geisler, Gry Aarum Geitvik, Solveig Hofvind, Vessela N Kristensen, Rolf Kåresen, Anita Langerød, Ole Christian Lingjærde, Gunhild Mari Mælandsmo, Hege G Russnes, Torill Sauer, Helle Kristine Skjerven, Ellen Schlichting, Therese Sørlie, Tone F. Bathen, Guro F. Giskeødegård

**Affiliations:** 1 Department of Public Health and Nursing (ISM), Norwegian University of Science and Technology- NTNU, Trondheim, Norway; 2 Department of Circulation and Medical Imaging, NTNU, Trondheim, Norway; 3 Department of Oncology, Division of Cancer Medicine, Oslo University Hospital, Oslo, Norway; 4 Institute of Clinical Medicine, University of Oslo, Oslo, Norway; 5 Vestre Viken Hospital Trust, Oslo, Norway; 6 Oslo University Hospital, Oslo, Norway; 7 Østfold Hospital, Oslo, Norway; 8 Akershus University Hospital, Oslo, Norway; 9 Cancer Registry of Norway, Oslo, Norway; 10 University of Oslo, Oslo, Norway

**Keywords:** Breast cancer, Cancer epidemiology

## Abstract

Breast cancer (BC) is a leading cause of cancer-related death worldwide. The diverse nature and heterogeneous biology of BC pose challenges for survival prediction, as patients with similar diagnoses often respond differently to treatment. Clinically relevant BC intrinsic subtypes have been established through gene expression profiling and are implemented in the clinic. While these intrinsic subtypes show a significant association with clinical outcomes, their long-term survival prediction beyond 5 years often deviates from expected clinical outcomes. This study aimed to identify naturally occurring long-term prognostic subgroups of BC based on an integrated multi-omics analysis. This study incorporates a clinical cohort of 335 untreated BC patients from the Oslo2 study with long-term follow-up (>12 years). Multi-Omics Factor Analysis (MOFA+) was employed to integrate transcriptomic, proteomic, and metabolomic data obtained from the tumor tissues. Our analysis revealed three prominent multi-omics clusters of BC patients with significantly different long-term prognoses (*p* = 0.005). The multi-omics clusters were validated in two independent large cohorts, METABRIC and TCGA. Importantly, a lack of prognostic association to long-term follow-up above 12 years in the previously established intrinsic subtypes was shown for these cohorts. Through a systems-biology approach, we identified varying enrichment levels of cell-cycle and immune-related pathways among the prognostic clusters. Integrated multi-omics analysis of BC revealed three distinct clusters with unique clinical and biological characteristics. Notably, these multi-omics clusters displayed robust associations with long-term survival, outperforming the established intrinsic subtypes.

## Background

With an estimated 2.3 million new cases worldwide, female breast cancer (BC) became the most diagnosed cancer type among women globally in 2020, accounting for 11.7% of all cancer cases [1]. Despite the recent decline in death rates, BC is still a leading cause of cancer death [1]. BC’s diverse nature and heterogeneous biology impose challenges in assessing prognosis as patients with similar clinical subtypes often experience different responses to treatment [2]. These challenges engendered many studies to probe BC at the molecular level in aspiration to reveal new potential treatment targets and clinically relevant prognostic clusters. Perou et al. characterized BC based on the gene expression variations in human breast tumors, revealing the prominent subtypes basal-like, luminal, Erb-B2+ (Her2 enriched), and normal-like- with distinctive molecular portraits [3]. The luminal subtype was subsequently divided into more refined groups -luminal A, B and C [4]. These molecular subtypes showed a strong association with clinical outcomes; patients with basal-like tumors had the worst prognosis while patients with the luminal A (LumA) subtype had the best prognosis [3–5]. These BC subtypes were later classified by gene expression of 50 discriminatory genes called prediction analysis of microarrays 50 (PAM50) and are now implemented clinically to classify BC intrinsic subtypes [6].

Studies have shown heterogeneity within the PAM50 transcriptomic-based intrinsic subtypes, suggesting refined subclusters within the subtypes that better explain patient survival [7, 8]. Although luminal-like subtypes generally have a good prognosis, the LumA subtype itself exhibits significant biological and clinical heterogeneity [9]. The prognostic value of the PAM50 intrinsic subtype for long-term survival above 10 years has shown varying results. For instance, studies suggest that luminal B (LumB) exhibits a poorer long-term prognosis than basal, particularly evident after a 10-year follow-up [10, 11]. Characterizing BC in a more comprehensive biological manner may improve patient stratification, the assessment of long-term prognosis, and optimal treatment. Investigating the mRNA expression alone for the characterization of BC may not capture the full picture as several studies report low correlations between the expression level of mRNA and tumor-related proteins [12–14]. For instance, in a study quantifying 52 BC-related proteins, only 22 proteins were reported to correlate with the level of transcripts [14]. The observed limited protein-mRNA correlations deliver substantial rationale to incorporate proteins in the BC characterization. As a result, six clinically relevant BC subtypes have already been described using reverse phase protein arrays (RPPA) [15]. Among these subtypes, four subgroups remained highly concordant with PAM50 subtypes and are consequently named Luminal A, Luminal B, Basal and Her2 rich. Further, two additional subgroups, reactive I and reactive II, were described as products of the surrounding microenvironment [16].

Metabolites are small molecules downstream to proteins, RNA and DNA, thus reflecting intermediates and endpoints of biological pathways. Metabolomics provides unique pathophysiological signatures influenced by gene variability and the surrounding environment, thus revealing information that is closely associated with the phenotype. Altered metabolism is described as a hallmark of cancer [17]. We have previously reported metabolic variation between BC tumors and normal adjacent tissue, and metabolic patterns related to clinical and prognostic factors of BC [18–20]. Furthermore, three distinct clusters of BC patients were reported based on tumor metabolic profiles, where the clusters expressed differences in BC-related proteins and genes, particularly those associated with the extracellular matrix and cancer-related metabolic pathways [21].

**Fig. 1:**
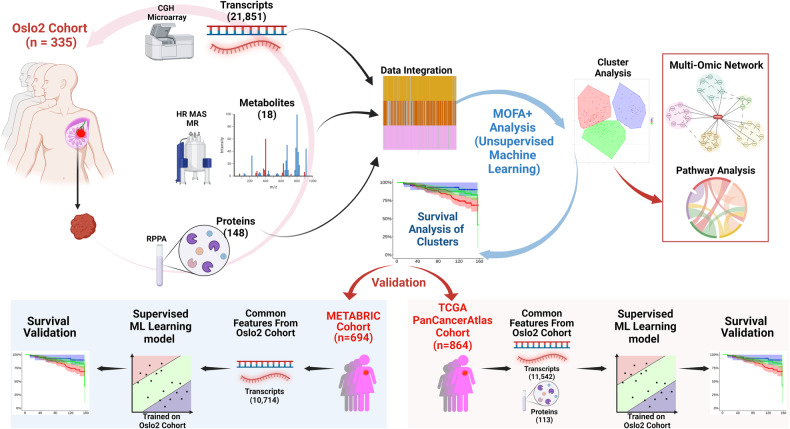
A graphical overview of the study framework. Transcriptomic, metabolomic and proteomic profiles were obtained from BC tissue samples of 335 patients within the Oslo2 cohort [21]. These multi-omics modalities were analyzed through an integrative unsupervised machine learning approach, Multi-Omics Factor Analysis (MOFA+), followed by a clustering analysis identifying three multi-omics clusters. The formation of clusters was based on the survival-associated latent factors identified by the MOFA+ model. A system biology approach was performed to further characterize these newly identified clusters. Finally, the multi-omics clusters were validated in large publicly available cohorts -TCGA (PanCancer Atlas) and METABRIC- using a supervised machine learning framework.

Based on previous molecular characterizations of BC tissue, it is evident that a global assessment of biomolecules across different omics modalities may provide deeper insights into the heterogeneity of BC, thus improving the prediction of long-term survival. Integration of multi-omics profiles has already revealed clinical subtypes of several heterogeneous cancers including colon cancer, urothelial bladder cancer and esophageal carcinoma [22–25]. Despite these recent advancements in molecular research techniques, a limited number of comprehensive studies have been reported that utilize multi-omics data to probe BC heterogeneity [26]. In this study, we aimed to categorize BC patients into clinically relevant clusters that improve prediction of long-term survival, based on extensive biological information rather than single molecular levels. We hypothesized that multi-omics data provides a more holistic view of the molecular patterns of BC thus providing a clinically credible assessment during longer follow-ups. Using unsupervised machine learning to integrate transcriptomic, proteomic and metabolomic data from tumor tissues of BC patients prior to adjuvant therapy, we revealed three prominent clusters of BC patients with significant differences in long-term survival beyond 10 years. These findings were validated with high consistency in large cohorts of BC patients with long-term follow-up (Fig. 1) (Table 1). Moreover, we unmasked the key characterizing pathways and possible metastasis drivers through a systems-biology approach, ultimately providing deeper molecular insights into the bio-mechanisms underlying BC heterogeneity.

**Table 1:** Clinical characteristics of the patient cohorts.

Cohort	Discovery Cohort	Validation Cohorts	
	Oslo2	TCGA PanCancer Atlas	METABRIC
Number of patients included in this study	335	859	694
Age (years), Mean (±SD)	56.16 (11.01)	58.05 (13.3)	61.08 (12.9)
Cancer Grade *n* (%)			
I	49 (14.6)	128 (14.9)	80 (11.5)
II	130 (38.8)	499 (58.1)	319 (46.0)
III	147 (43.9)	206 (24.0)	250 (36.0)
IV	0	16 (1.9)	0
NA or X	9 (2.7)	10 (1.2)	45 (6.5)
Node status *n* (%)			
pN0	202 (60.3)	404 (47.0)	*–*
pN1(mi)	10 (3.0)	28 (3.3)	*–*
pN1	83 (24.8)	250 (29.1)	*–*
pN2	21 (6.3)	100 (11.6)	*–*
pN3	11 (3.3)	64 (7.5)	*–*
NA	8 (2.4)	13 (1.5)	*–*
Primary tumor *n* (%)			
pTis	9 (2.7)	0	*–*
pT1	176 (52.5)	197 (22.9)	*–*
pT2	123 (36.7)	516 (60.1)	*–*
pT3	15 (4.5)	114 (13.3)	*–*
pT4	0	31 (3.6)	*–*
NA	12 (3.6)	1 (0.1)	*–*
Histology *n* (%)			
Ductal	269 (80.3)	624 (72.6)	504 (72.6)
Non-Ductal	58 (17.3)	234 (27.2)	184 (26.5)
NA	8 (2.4)	1 (0.1)	6 (0.9)
HR + HER- *n* (%)	238 (71.0)	*–*	567 (81.7)
HER2 ( + /-/*NA*) *n* (%)	34/284/17 (10.1/84.7/5.0)	*–*	63/631/0 (9.7/90.9/0)
TNBC (Yes/No/*NA*) *n* (%)	46/272/17 (13.7/81.1/5.0)	*–*	64/630/0 (9.2/90.7/0)
Intrinsic Subtype *n* (%)			
Basal-like	55 (16.4)	143 (16.6)	45 (6.5)
HER2 enriched	48 (14.3)	71 (8.3)	71 (10.2)
Luminal A	79 (23.6)	385 (44.8)	301 (43.4)
Luminal B	72 (21.5)	166 (19.3)	163 (23.5)
Normal-like	53 (15.8)	27 (3.1)	60 (8.6)
Claudin-low	*–*	*–*	50 (7.2)
NA	28 (8.4)	67 (7.8)	4 (0.6)
Death *n* (%)	82 (24.4)	120 (14.0)	449 (64.7)
BC-specific	40 (11.9)	65 (7.5)	203 (31.2)
Other Causes	26 (7.7)	37 (4.3)	246 (37.9)
Unknown cause	16 (4.7)	18 (2.0)	0
NA	4 (1.2)	0	0

(--) Unmeasured attributes for the cohort,

*HR* Hormone Receptor, *HER2* Human epidermal growth factor receptor 2, *TNBC* Triple Negative Breast Cancer, *(+)* positive, *(−)* Negative, *SD* standard deviation, *NA* not available.

## Results

### Multi-omics integration revealed molecular characteristics associated with clinical factors

**Fig. 2:**
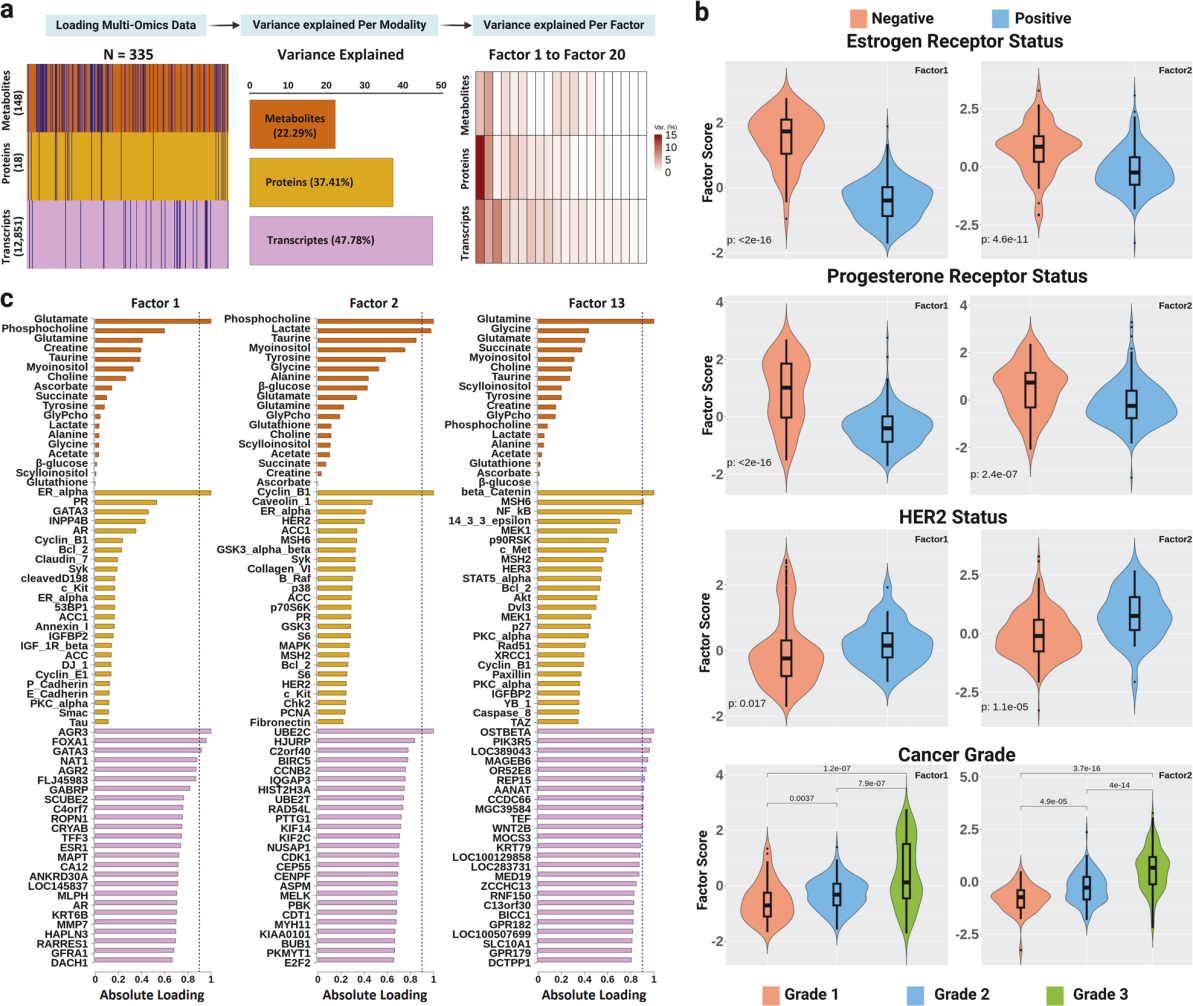
MOFA+ analysis of the Oslo2 cohort. **a** This illustration depicts the chronological steps involved in MOFA+ analysis. Once the multiple modality dataset is loaded, the MOFA model compresses the multi-omics data into 20 latent factors. This process also assesses the contribution of each modality and its corresponding factors in explaining variance. Multi-omics dataset layers and the summary is illustrated (left) followed by the total variance explained per modality (middle), and the proportion of variance explained by individual factors (right). The dark blue lines in the left plot indicate missing values. **b** The violin plots show the distribution of ER, PR, HER2 status and cancer grade for multi-omic factors 1 and 2. The *p* values are reported from two-sample Wilcoxon rank sum tests. In all panels, the center line of the boxplot represents the median, while the bounds of the box represent the interquartile range (IQR). **c** The absolute loadings of features in factor 1, factor 2 and factor 13 are displayed. The list includes all 18 metabolites, while only the top 25 most significant features from the proteome and transcriptome layers are shown. All absolute loadings for the latent factors are provided in Supplementary Data 2. The blue dotted vertical line represents the threshold for highly important targets (>0.9) within each modality. GlyPcho Glycerophosphocholine.

Trained on the Oslo2 data, the MOFA+ model with 20 latent factors provided a favorable balance between the average variance inflation factor (VIF) and the total variance explained by the model (Fig. 2a) (Supplementary Fig. 1). Factors 1 and 2 explained most of the molecular heterogeneity (25.7% and 16.2%, respectively). Interestingly, factor 1 captured higher variance through the protein expressions than via mRNA expressions, followed by metabolomics data (Fig. 2a). The Spearman’s rank correlation between clinical features of the tumor (tumor grade, histology, tumor size, lymph node status, hormone receptor status) and multi-omics factors (MOFs) is illustrated in Supplementary Fig. 2 (Supplementary Data 1). Factor 1 and 2 showed a significant correlation with the clinical characteristics of the tumor. (Fig. 2b) (Supplementary Data 1, Supplementary Fig. 2). Factor 1 showed a significant association with tumor grade (Kruskal–Wallis *p* = 8.1e-10). Factor 2 exhibited a strong association with tumor grade (Kruskal–Wallis *p* = 7.9e-22), histology (Kruskal–Wallis *p* = 1.9e-07) and size (Kruskal–Wallis *p* = 0.017) (Supplementary Data 1). The absolute loadings for each latent factor reveal important biological features that are responsible for data variability. We observed that estrogen receptor alpha (ERα), progesterone receptor (PR) and GATA binding protein-3 highly influenced factor 1 (Fig. 2c). Further, AGR3, FOXA1 and GATA3 genes were some of the most important gene targets underlying the variability captured by factor 1. The important metabolites according to factor 1 were glutamate (Glu), phosphocholine (pCho) and glutamine (Gln). The biological signals captured by factor 2 remained moderately even from all three modalities. The variance captured by metabolites was higher in factor 2 compared to factor 1 with pCho, lactate (Lac) and taurine (Tau) as important metabolites. Factor 2 highlighted Cyclin B1, Caveolin-1, and ERα as crucial proteins, while the genes UBE2C, HJURP, and C2orf40 were identified as significant contributors to the total variance explained. Interestingly, Factor 13 also showed a significant correlation with body mass index (BMI) and tumor size (Supplementary Fig. 2).

### Multi-omics clusters of breast cancer are related to long-term survival

**Fig. 3:**
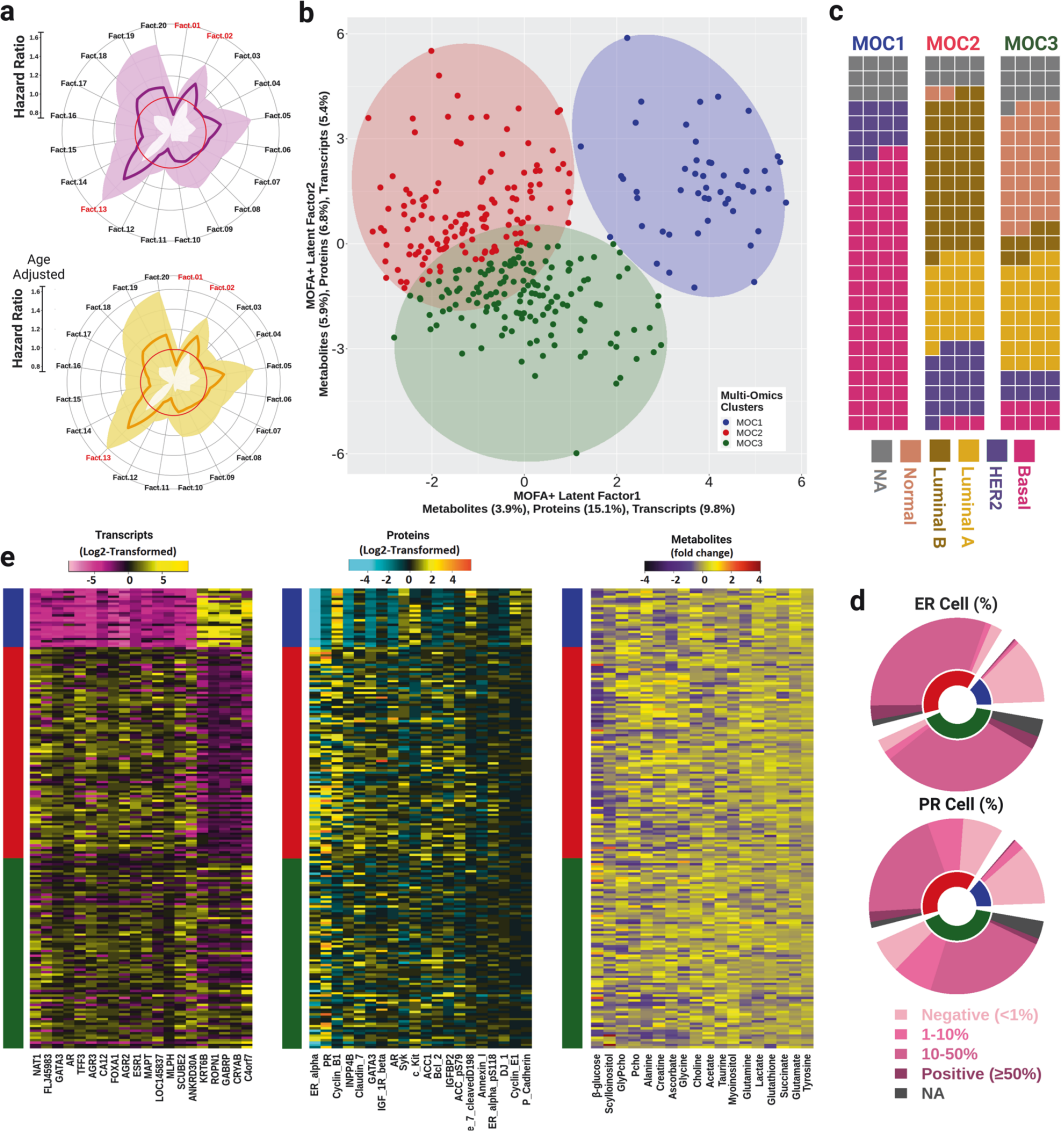
Molecular characterization of the multi-omics clusters. **a** The circular plot shows the hazard ratio (HR) of breast cancer-specific death and/or metastasis derived from a multivariate Cox regression model fitted on the set of 20 MOFs (upper), while also adjusting for age (bottom). The bold line presents the HR of the factors. The red circle marks the line of null effect. The shaded region shows the 95% confidence intervals. Factors 1, 2 and 13 highlighted in red are significantly associated with survival (Wald test *P* < 0.05). **b** By employing k-means clustering, the combination of BC-specific survival and metastasis-explaining factors (1,2 and 13) led to the formation of three multi-omics clusters (MOCs) -MOC1, MOC2 and MOC3- for the MOFA+ model. **c** The waffle chart elucidates the relative proportions of intrinsic subtypes within each MOCs. Each MOC is depicted as a block employing 25 × 4 grids, with each cell representing 1%,(totaling 100%). **d** The relative distribution of hormone receptors level (outer layer) within each MOCs (inner layer) is illustrated in the multi-level pie chart. The upper chart depicts ER level, while the lower chart shows the PR level. The MOC color keys follow from **b**. **e** The heat map of the expression levels of the top 20 MOFA+ derived significant transcripts, proteins, and all 18 metabolites.

To assess the prognostic value of the MOFA+ model, multivariate Cox proportional-hazards models were constructed and hazard ratios (HR) with confidence intervals (CI) are reported. In the Oslo2 cohort, 62 patients died due to BC or had BC metastasis during the follow-up period (average time to event = 9.5 years, SD = ± 3.5 years). Crude Cox regression analyses revealed that factor 1 (HR 0.84, 95% CI: 0.72–0.97, *P* = 0.021), factor 2 (HR 1.18, 95% CI: 1.00–1.39, *P* = 0.045), and factor 13 (HR 1.29, 95% CI: 1.05–1.58, *P* = 0.015) were significantly associated with metastasis and/or BC specific death (Fig. 3a). Adjusting for age did not substantially alter the hazard ratios of these factors. Forest plots for the Cox regression are provided in Supplementary Fig. 3.

Clustering based on MOFs 1,2 and 13 revealed three multi-omics clusters (MOC) (Fig. 3b). These clusters -MOC1, MOC2, and MOC3- included 50, 137 and 148 patients, respectively. An overview of clinical information for each cluster is presented in Table 2. MOC1 demonstrated a predominant enrichment of the basal-like subtype (74%) and a smaller presence of HER2-enriched samples (14%), with no other intrinsic subtypes observed (Fig. 3c). MOC2 contained a mixture of LumB (42%), LumA (26%) and HER2 (20%). The largest cluster MOC3 was highly enriched with LumA (29%) and Normal type (34%) with few basal, HER2 and LumB type samples. In addition, the distribution of ER, PR and HER2 hormone receptor statuses (measured by immunohistochemistry) was noticeably different between the MOCs (Fig. 3d). As expected, the MOCs varied in the pattern of expression in transcriptomic, proteomic, and metabolomic profiles (Fig. 3e). Key breast cancer-associated biomarker genes such as CRYAB and GABRP showed high upregulation in MOC1, while they remained lowly expressed in MOC2 and displayed intermediate expression levels in MOC3 (Supplementary Data 3) [27]. MOC1 exhibited a significant downregulation of GATA3 (Kruskal–Wallis test, *P*_adj_ = 8.3e-22), AGR3 (*P*_adj_ = 4.0e-20) and FOXA1 (*P*_adj_ = 7.0e-22) compared to the other MOCs. In the protein expression profile, MOC1 had upregulated Cyclin B1 (*P*_adj_ = 5.3e-22). MOC1 demonstrated significantly low expression levels of ER-alpha (*P*_adj_ = 1.01e-21) and PR (*P*_adj_ = 5.9e-15) proteins, consistent with the low proportion of hormone receptor-positive cells in this cluster (Fig. 3d, e). The metabolite level of alanine (*P*_adj_ = 4.8e-03), β-glucose (*P*_adj_ = 5.8e-07), taurine (*P*_adj_ = 4.8e-03), lactate (*P*_adj_ = 8.0e-06) and glutamate (*P*_adj_ = 1.6e-09) were significantly different between the MOCs (Supplementary Data 3). MOC3 exhibited a higher expression of β-glucose, while MOC2 displayed a high level of glutamine expression (Fig. 3e).

**Table 2:** Clinical characteristics of the multi-omics defined clusters in the study cohorts.

Cohort	Discovery Cohort			Validation Cohorts					
	Oslo2			TCGA			METABRIC		
	MOC1	MOC2	MOC3	MOC1	MOC2	MOC3	MOC1	MOC2	MOC3
Number of patients	50	137	148	133	402	324	42	341	311
Age (years), mean (±SD)	55.9 (12.3)	56.9 (11.4)	55.9 (9.8)	55.3 (12.7)	59.6 (13.9)	57.3 (12.6)	60.9 (12.3)	66.7 (11.4)	62.9 (12.6)
Cancer Grade, *n* (%)									
I	0	14 (10.2)	35 (23.6)	13 (9.7)	59 (14.68)	56 (17.2)	0	27 (7.9)	53 (17.0)
II	0	57 (41.6)	73 (49.3)	97 (72.9)	231(57.4)	171 (52.7)	3 (7.1)	158 (46.3)	158 (50.8)
III	50 (100)	63 (46.0)	34 (23.0)	19 (14.2)	96 (23.8)	90 (27.7)	39 (92.9)	133 (39.0)	78 (25.1)
IV	0	0	0	1 (0.75)	11 (2.7)	4 (1.2)	0	0	0
NA	0	3 (2.2)	6 (4.1)	3 (2.26)	5 (1.24)	2 (0.62)	0	23 (6.7)	22 (7.1)
Lymph Node status, *n* (%)									
pN0	40 (80)	69 (50.4)	93 (62.8)	84 (63.1)	172 (42.7)	146 (45.0)	--	--	--
pN1(mi)	1 (2)	5 (3.6)	4 (2.7)	2 (1.5)	10 (2.49)	16 (4.9)	--	--	--
pN1	6 (12)	42 (32.1)	31 (20.9)	32 (24.06)	132 (32.8)	86 (26.5)	--	--	--
pN2	2 (4)	9 (6.6)	10 (6.8)	10 (7.52)	52 (12.9)	38 (11.73)	--	--	--
pN3	1 (2)	7 (5.1)	3 (2.0)	5 (3.76)	25 (6.2)	34 (10.4)	--	--	--
NA	0	3 (2.2)	5 (3.4)	0	9 (2.24)	4 (1.23)	--	--	--
Tumor size, *n* (%)							--	--	--
pTis	1 (2)	0	8 (5.4)	0	0	0	--	--	--
pT1	29 (58)	65 (47.4)	82 (55.4)	20 (15.04)	90 (22.3)	87 (26.8)	--	--	--
pT2	19 (38)	61 (44.5)	43 (29.1)	96 (72.18)	244 (60.7)	176 (54.3)	--	--	--
pT3	1 (2)	5 (3.6)	9 (6.1)	12 (9.02)	49 (12.1)	53 (16.3)	--	--	--
pT4	0	0	0	5 (3.76)	19 (4.73)	8 (2.5)	--	--	--
NA	0	6 (4.4)	6 (4.1)	0	1 (0.25)	0	--	--	--
Histology *n* (%)									
Ductal	47 (94)	118 (86.1)	104 (70.3)	120 (90.2)	327 (81.3)	176 (54.3)	36 (85.7)	267 (78.3)	201 (64.6)
Non-Ductal	3 (6)	16 (11.7)	39 (26.4)	12 (9.02)	75 (18.6)	148 (45.7)	5 (11.9)	71 (20.8)	108 (34.7)
NA	0	3 (2.2)	5 (3.4)	1 (0.75)	0	0	1 (2.4)	3 (0.9)	2 (0.6)
Metastasis *n* (%)									
Yes	3 (6)	35 (25.5)	22 (14.8)	2 (1.5)	13 (3.2)	7 (2.1)	--	--	--
No	45 (90)	102 (74.4)	124 (83.7)	114 (85.7)	350 (87)	255 (78.7)	--	--	--
NA	2 (4)	0	2 (1.3)	17 (12.7)	39 (9.7)	62 (19.1)	--	--	--
Recurrence *n* (%)									
Yes	8 (16.0)	46 (33.5)	29 (19.5)	14 (10.5)	26 (6.4)	44 (13.5)	19 (45.2)	111 (32.5)	129 (41.2)
Maybe	0	0	3 (2)	--	--	--	--	--	--
No	43 (86)	125 (91.2)	134 (90.5)	107 (80.4)	312 (77.6)	256 (79)	23 (54.7)	230 (67.4)	182 (58.5)
NA	2 (4)	1 (0.7)	4 (2.7)	12 (9)	64 (15.9)	24 (7.4)	--	--	--
HR + HER2- *n* (%)	4 (8.0)	113 (82.4)	121 (81.7)	--	--	--	4 (9.5)	292 (85.6)	271 (87.1)
HER2 ( + /-/NA) *n* (%)	6/43/1 (12/86/2)	20/114/3 (14.5/83.2/2.1)	8/127/13 (5.4/85.8/8.7)	--	--	--	4/38/0 (9.5/90.4/0)	26/315/0 (7.6/92.3/0)	33/278/0 (10.6/89.3/0)
TNBC (Yes/No/NA) *n* (%)	39/10/1 (78/20/2)	1/133/3 (0.7/97/2.1)	6/129/13 (4.0/87.1/8.7)	--	--	--	35/7/0 (83.3/16.6/0)	10/331/0 (2.9/97/0)	19/292/0 (6.1/93.9/0)
Death *n* (%)	6 (12)	47 (28.5)	33 (16.9)	16 (12.0)	68 (16.9)	36 (11.1)	21 (50.0)	236 (69.2)	192 (61.7)
BC-specific	2 (4)	23 (16.78)	15 (10.1)	9 (6.8)	35 (8.7)	21 (6.5)	10 (23.8)	106 (31.1)	87 (27.9)
Other Causes	2 (4)	15 (10.94)	9 (6.0)	3 (2.2)	21 (5.2)	13 (4.0)	11 (26.2)	130 (38.1)	105 (33.7)
Unknown cause + NA	2 + 0 (4)	8 + 1 (6.56)	6 + 3 (6.0)	4 (3.0)	12 (3.0)	2 (0.6)	0	0	0

(--) Unmeasured attributes for the cohort.

*HR* Hormone Receptor, *HER2* Human epidermal growth factor receptor 2*, TNBC* Triple Negative Breast Cancer*, (+)* positive, *(−) Negative, SD* standard deviation*, NA* not available*.*

**Fig. 4:**
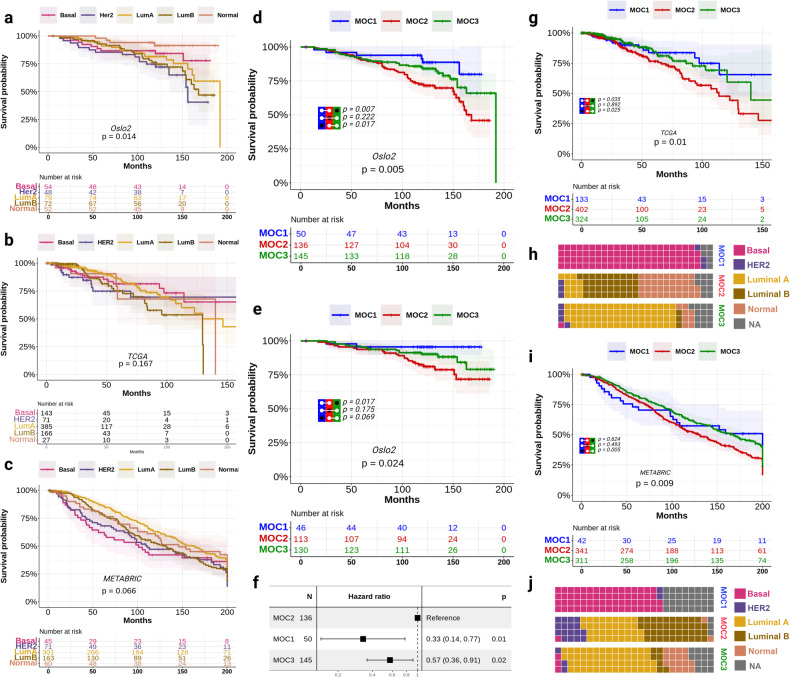
Survival analysis of the multi-omics clusters and validation of the multi-omics clusters in external cohorts. The Kaplan–Meier curves show overall long-term survival for different intrinsic subtype groups for all-cause mortality in **a** Oslo2, **b** TCGA and **c** the METABRIC cohort. The Kaplan–Meier curves show **d** overall survival for the different MOC groups and **e** BC-related death respectively. The log-rank *p*-value is inscribed on the plots. *P*-values from the log-rank test of the three clusters in addition to the pairwise log-rank test between MOCs are shown. The risk table is illustrated below the curves. **f** The forest plot illustrates hazard ratios (HR) of all cause mortality where MOC2 is the reference group, in comparison to MOC1 and MOC3 for the Oslo2 cohort. The estimated HR is represented by a box and the whiskers indicate the 95% confidence intervals. On the right side, Wald test *p*-values are shown. The Kaplan–Meier curves illustrate the overall long-term survival of MOCs in **g** TCGA and **i** the METABRIC cohort. *p*-values from the log-rank test of the three clusters in addition to pairwise log-rank test between MOCs are shown. **h**, **j** The waffle chart elucidates the relative proportions of intrinsic subtypes within each MOCs of TCGA and METABRIC respectively.

Interestingly, the investigation of long-term prognosis among intrinsic subtypes for the Oslo2 cohort yielded outcomes that differed from the expected clinical patterns. On a longer follow-up (~120 months), the luminal subtypes demonstrated an unexpectedly poor prognosis when compared to the basal-like subtypes, defying the expected clinical pattern (Fig. 4a). Such anomalous observations were also seen in the TCGA and METABRIC cohorts when the long-term survival of intrinsic subtypes was accessed (Fig. 4b, c), where the long-term survival of the intrinsic subtypes was not significantly different.

There was a significant difference in the overall survival rates between the MOCs (log-rank test *p* = 5e-03) when assessing the long-term survival up to 15 years after surgery. (Fig. 4d). MOC1 exhibited a good prognosis compared to MOC2 while remaining moderately better than MOC3. Further, similar survival curves between the MOCs were observed when only death due to BC was examined (log-rank test *p* = 0.024) (Fig. 4e). MOC2 had the poorest prognosis compared to MOC1 and MOC3 (Fig. 4f). Significant differences in outcome between the MOCs were also demonstrated when investigating the cohort with (a) BC-specific death and/or metastasis and, (b) BC-specific death and/or metastasis including the relapse event (Supplementary Fig. 4). MOC2 comprises more metastasis and relapse events compared to the other MOCs.

### Validation of the multi-omics clusters in external cohorts

To validate the differences in survival between the MOCs, a machine-learning (ML) based validation scheme was used for each external cohort separately i.e., TCGA and METABRIC. The common transcriptomes or/and proteins between the validation cohorts and Oslo2 cohort were identified (Supplementary Data 4). These omics features were then used to train the ML ensemble to classify the MOCs of the Oslo2 cohort. Classification results for the models are presented in Supplementary Data 5–6. The derived models were then used to classify samples from the validation cohorts as MOC1, 2 or 3 (Table 2).

For the TCGA cohort, the all-cause survival rates between the MOCs remained highly concordant with the Oslo2 cohort, where MOC1 had a good prognosis while MOC2 depicted the worst prognosis (Fig. 4g). The gene expression profiles of MOCs in TCGA and Oslo2 cohorts remained highly congruent. Similar to the MOC1 in Oslo2, the MOC1 in the TCGA cohort had high enrichment of the Basal-like subtype and few HER2-enriched subtypes (Fig. 4h). Similarly, MOC3 in TCGA remained highly enriched for the LumA and Normal subtypes. MOC2 in TCGA remained predominantly enriched by LumB as for MOC2 from Oslo2. However, the MOC2 samples in TCGA also consisted of samples of Normal subtypes.

In the METABRIC cohort, the MOCs had significant differences in the long-term all-cause survival rates with survival trends similar to those found in the Oslo2 cohort. Initially, the prognosis of MOC1 was the poorest; however, it gradually improved compared to MOC2 during the follow-up period. (Fig. 4i). The PAM50 enrichment of MOCs was also similar to Oslo2, where MOC1 was basal rich while the enrichment of MOC2 and MOC3 were particularly conformable with the Oslo2 cohort’s MOCs (Fig. 4j). Similar observations were also found for both cohorts when only the BC-specific mortality was assessed (Supplementary Fig. 5). The clinical features of MOCs across the validation cohort are presented in Table 2. The proportion distribution of these features between the MOCs remained consistent across all the cohorts.

These validation results remained highly consistent also when the analysis encompassed all the samples, irrespective of their history of receiving neoadjuvant therapy (Supplementary Fig. 6).

### Functional analysis

Functional analyses were performed to determine differentially expressed pathways between the multi-omics clusters. The analysis was focused on MOC1 and MOC2 because of their large difference in prognosis. Two class significance analysis of microRNA (SAM) method revealed 9450 differentially expressed genes (DEGs) between MOC1 and MOC2 at false discovery rate (FDR) of 0.01 (Supplementary Fig. 7) (Supplementary Data 7). The KEGG database-based gene set enrichment analysis (GSEA) revealed the upregulation of prominent pathways related to cytokine-cytokine receptor interactions in MOC1 (KEGG ID: hsa04060, hsa04061, hsa04650) (Supplementary Fig. 8). The gene ontology (GO) MSigDB (C5) database suggested the upregulation of pathways related to the immune system (GO: 0002684, 0045087, 0002250) in MOC (Supplementary Fig. 8). In addition, the GSEA based on MSigDB (C6) database showed upregulation of Raf1 pathways in MOC2 (Supplementary Fig. 8)

**Fig. 5:**
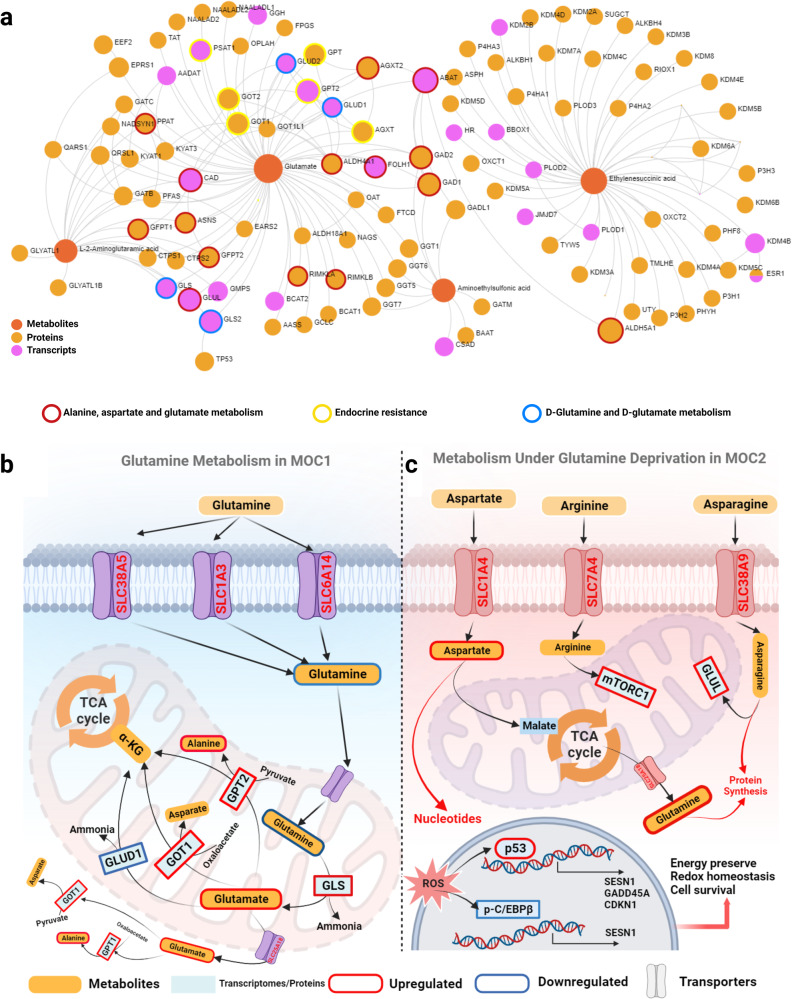
Systems-biology analysis comparing MOC1 and MOC2. **a** Multi-omics pathway analysis incorporating the differentially expressed multi-omics features -transcriptomics, proteomics and metabolomics. The network topology including the enriched multi-omics pathways and the corresponding scores are provided in Supplementary Data 8. **b** MOC1 has upregulated glutamine transporters such as SLC1A5, SLC38A1/SLC38A2, and SLC6A14. These transporters are located on the cell membrane. Once inside the cell, the SLC1A5 transports glutamine to the mitochondrial matrix. The high expression of GLS in MOC1 implicates the glutaminolysis within the mitochondrial matrix. The glutamate derived from glutamine is then catalyzed into α-KG by the GLUD1, GOT2, and GPT2 enzymes. This conversion leads to the release of ammonia, aspartate, and alanine, respectively, and largely contributes to the maintenance of redox homeostasis. **c** Under glutamine deprivation in the MOC2 tumor cells, p53, a tumor suppressor protein upregulated in MOC2, triggers the expression of SLC1A4 and SLC7A4 transporters. SLC1A4 facilitates aspartate uptake and could lead to increased malate levels, an intermediate in the TCA cycle. This, in turn, may amplify oxidative phosphorylation and glutamine synthesis. Aspartate is also utilized for nucleotide synthesis. Furthermore, the possible mediation of arginine through the upregulated SLC7A4 transporter in MOC2 may explain the high expression of mTORC1 in MOC2, a protein suppressed during glutamine depletion. The intracellular asparagine levels rise also implicate the higher expressions of GLUL proteins in MOC2, leading to heightened glutamine and protein synthesis. The upregulation of p53 target genes (SESN1, GADD45A and CDKN1), as well as the phosphorylation of C/EBPβ and its target gene (SESN2) helps maintain energy and redox balance, ultimately promoting cancer cell survival in MOC2. GLS glutaminase, α-KG α-ketoglutarate, GLUD1 glutamate dehydrogenase 1, GOT glutamate oxaloacetate transaminase, GPT glutamate pyruvate transaminase, TCA tricarboxylic acid cycle, ROS reactive oxygen species, GLUL glutamate-ammonia ligase, C/EBPβ CCAAT/enhancer binding protein β. Pathways adapted from Jin, J et al. [34].

Multi-omics pathway analysis was carried out by incorporating the differentially expressed omics features between MOC1 and MOC2. The network generated through metabolite-protein interactions and protein-protein interactions were merged to form a multi-omics network through shared nodes (Fig. 5a). The network comprises 4 metabolites and 114 mRNA/proteins. The network matrix and detailed topology are provided in Supplementary Data 8. To further explore the functional pathways, three KEGG databases were referred including KEGG (Metabolites), KEGG (Gene + Metabolites) and KEGG (gene/protein) to highlight the nodes from the enriched pathways. The analysis identified aberrations in alanine, aspartate and glutamate metabolism (*P*_adj_ = 1.07e-38), Endocrine resistance (*P*_adj_ = 6.42e-9), and the D-glutamine and D-glutamate pathways (*P*_adj_ = 1.78e-6) between the MOC1 and MOC2 clusters (Supplementary Data 8).

## Discussion

The heterogeneity of BC is rooted in the biomolecular variations present among the cancer cells. These variations affect tumor characteristics such as therapeutic responses and metastasis. RNA-based intrinsic subtyping of BC is an established approach for BC characterization that focuses on inter-tumor heterogeneity. However, several studies suggest that the established intrinsic subtyping does not emphasize the intra-tumor admixture of multiple subtypes leading to imprecise subtyping [28, 29]. We here show that the intrinsic subtyping based on the PAM50 gene set distinctly demonstrates long-term survival outcomes that are contrary to the expected clinical prognosis, for the Oslo2 cohort, but also in the large validation cohorts METABRIC and TCGA. Patients with a basal-like subtype showed relatively good long-term prognosis compared to the remaining subtypes, while the luminal type suffered poorer survival outcomes. In addition, we have previously observed that LumA tumors often have a delayed local relapse and metastasis when compared to other subtypes [30]. We observe a similar trend in our analysis (Supplementary Fig. 4). These observations challenge the conventional assumptions about the clinical nature of these intrinsic subtypes. Earlier research has corroborated these findings, wherein some studies attempted to reclassify intrinsic subtypes while providing plausible explanations for the observed outcomes [7, 8]. To address these challenges in assessing long-term prognosis, it is crucial to incorporate multiple omics modalities to capture the deep-embedded biological heterogeneity. Using this approach, we revealed three new clusters of BC patients with significantly different long-term survival rates. The survival trends between the clusters were further validated in the external cohorts -TCGA and METABRIC- with high consistency. Moreover, the distribution of intrinsic subtypes and clinical variables exhibited significant variation between these diverse cohorts, highlighting the robustness of our clusters. This, in turn, may offer new grounds for enhanced characterization and understanding of BC. It is worth noting that even though metabolomics data were not available for the validation cohorts, we observed a clear survival curve separation between the MOCs, especially in the TCGA cohort. However, this does not necessarily imply that metabolic markers explain less biological heterogeneity compared to transcripts and protein markers. The metabolic data explained the lowest amount of variation in the discovery cohort; however, this may be attributed to high missing values and fewer variables in Oslo2 metabolomic data compared to the other omics data. As metabolic data were used to define the original MOCs, and supervised models were built to classify the validation cohorts based on available data, the metabolic data are likely to have influenced the MOCs also in the cohorts where metabolites are not directly measured, as there is a delicate interplay between metabolites, proteins and transcripts.

Of the three multi-omics clusters, MOC1 showed the best prognosis, while MOC2 had the worst. Interestingly, MOC1 is also highly rich in basal-type cancer which also commonly occurs in younger women [31]. However, the factors underpinning MOCs (i.e., Factor 1, Factor 2 and Factor 13), show a clear association with BC-specific survival even in age-adjusted models. This suggests intricate underlying biological causes other than age or clinical features for such observation in MOC1. Multi-omics pathway analysis suggested differences related to the D-glutamine and D-glutamate metabolism to be among the main molecular differences between the clusters. D-glutamine and D-glutamate metabolism has been studied for their role in endothelial cell regulation and potential role in the metastasis of BC [32]. Comparing the molecular characteristics of MOC1 and MOC2 (Fig. 3e) we observed that MOC2 had a significantly higher level of glutamine (Gln) and a lower level of glutamate (Glu) which is formed directly from Gln through the process of glutaminolysis. This indicates a high degree of altered glutamine metabolism between MOC1 and MOC2 where the former exhibits an increased level of glutaminolysis. The transcript level of glutaminase (GLS), the enzyme that catalyzes glutaminolysis, was significantly upregulated in MOC1 when compared to MOC2 and MOC3, thus supporting the hypothesis. Gln is a conditionally essential amino acid and Gln-metabolism is considered an important therapeutic target, especially since certain cancer cells exhibit heightened glutamine uptake and dependency [33, 34]. Through transporters such as SLC1A5 and SLC7A5, which were also found upregulated in MOC1, the Glu further gets converted into enzymes that enable ATP production via the TCA cycle which is necessary for cancer cell growth and survival (Fig. 5b, c) [35]. Furthermore, activation of alanine, aspartate and glutamate metabolism (KEGG pathway map00250) was observed in MOC2. This pathway is known to provide critical fuel for survivability and proliferation of cancer cells [36]. The proteomic characteristics of MOC2 include a high level of Cyclin-Dependent Kinase 1 (CDK1), CDK2 and Cyclin D protein. Upregulation of CDKs and Cyclin D proteins along with the phosphatidylinositol 3-kinase (PIK3) gene family in MOC2 indicate the activation of the PI3K/Akt/mTOR pathway [37, 38]. PI3K-AKT signaling pathway has been implicated in endocrine resistance [39]. Supporting this, multi-omics pathway analysis revealed the endocrine resistance pathway as a significant pathway (13 hits) when comparing MOC1 and MOC2.

Interesting differences between the omics characteristics of MOC3 and the other two MOCs were also observed. The concentration levels of Gln, Glu and Cho were significantly different between all clusters with an intermediate expression level of these metabolites in MOC3 when compared to MOC1 and MOC2. MOC3 tumors are also characterized by significantly higher concentration levels of taurine and moinositol compared to MOC1 and MOC2 suggesting high apoptosis in BC cells [40, 41]. Interestingly, the GSEA analysis support the increased apoptosis of tumor cells in MOC3 as pathways crucial for the mitotic cell cycle were significantly downregulated in MOC3 when compared to MOC2 (Supplementary Fig. 9). These observations may explain the low mortalities in MOC3 compared to MOC2, however, further investigation is warranted.

This study showed that the integrative multi-omics approach allowed the discovery of robust long-term prognostic MOCs, however, it is important to highlight the challenge this approach poses in terms of clinical practicality. Although incorporating more omics modalities in the framework could improve our understanding of BC heterogeneity and prognosis, it is uncommon to obtain such information at the individual level. However, as demonstrated through TCGA and METABRIC cohorts, not every level of omics is required for classifying MOCs. A robust supervised model, trained on a larger cohort, could attain clinically credible precision in MOC classification using a limited panel, similar to the PAM50. Furthermore, as the high-throughput technology continues to advance, integrating multi-omics into clinical practice may become feasible. One of the challenges in investigating long-term outcomes is that the treatment may be partly outdated during the long-term follow-up, especially for cancer where remarkable advancements in treatment regimens have been made in previous decades (endocrine treatment, chemotherapy including intensity, HER2 directed treatment, post-neoadjuvant treatment alterations). This may be reflected in our validation study where the curve separation in TCGA is in agreement with Oslo2, while the basal type rich MOC1 in the METABRIC cohort shows a different 5-year breast cancer mortality. This difference in outcomes may be attributed to the fact that TCGA and Oslo2 are relatively newer cohorts compared to METABRIC, consequently benefiting from more advanced and improved treatment strategies. However, it is also interesting to observe that MOC1 has 80% pN0 in Oslo2, versus 50% and 60% for MOC2 and MOC3 respectively (Table 2). In TCGA, 63% of patients are pN0 in MOC1 versus 43% and 45% in MOC2 and MOC3 respectively (Table 2). Since lymph node status is an important prognostic factor (Supplementary Fig. 3), this variation may also contribute to the favorable prognosis of patients in the MOC1 compared to MOC2/MOC3. It is worth noting that treatment information was not considered in our study, under the assumption that all patients received optimal treatment according to the disease type at the time of their diagnosis. Optimally, to obtain clinically reliable prognostic information from the proposed multi-omics approach, MOC subgroups should be compared within an independent patient cohort that has not received any systemic adjuvant treatment.

## Conclusion

Overall, the analysis presented in this study shows that integrated analysis of metabolomics, transcriptomics, and proteomics, can differentiate highly aggressive breast cancer from less aggressive types. Three prominent clusters of BC patients with different prognoses were revealed in this study, which was validated in two independent large cohorts with high consistency. Importantly, the multi-omics clusters showed robust associations with long-term survival compared to the established intrinsic subtypes. Using systems-biology approaches we unmasked possible key pathways and drivers that characterized the found multi-omics clusters, ultimately providing deeper molecular insights into the biological mechanisms underlying BC heterogeneity. Nonetheless, our study emphasizes the significance of the integrative multi-omics approach in future clinical studies of BC.

## Methods

### Oslo2 cohort

As a part of the Oslo2 study, tissue samples from untreated patients of primary breast carcinoma were collected at the Oslo University Hospital in the years 2006–2009 (Radium Hospital and Ullevål Hospital) [21]. Tumor tissue from a total of 335 patients were included in this study (Table 1). The obtained tissue samples were freshly frozen after surgery and stored at −80 °C. Further, the samples were dissected into three sections as previously described [21]. The two parts comprising the outer edges were used to perform histological evaluation including tumor cell percentage, estrogen receptor (ER) and progesterone receptor (PR) status. The middle section was used for metabolomics analysis, while the remnant of all three pieces was used for the extraction of DNA, RNA, and proteins.

Of 335 patients, metabolomics data, gene expression and protein expression profiles were available for 228, 308 and 315 samples, respectively. A complete dataset for all three omics profiles was available for 196 samples.

### Transcriptome profiling

Transcriptomics analysis was performed as previously described by Haukaas et al. [21]. In short, Total RNA was extracted with TRIzol. SurePrint G3 Human GE 8x60K (Agilent Technologies) was used to measure mRNA expression as per the manufactory’s protocol (one-color microarray-based gene expression analysis, low input Quick Amp Labeling, v.6.5, May 2010) and 100 ng RNA was used as input for labeling. Further, the obtained 21,851 profiles were quantile normalized, log2-transformed, and hospital-adjusted [21]. Measurements from the identical Entrez ID probes were averaged to create a single expression value per gene. Finally, intrinsic subtypes were assigned to each sample using the previously defined PAM50 algorithm [6].

### Protein expression

A total of 151 primary antibodies were probed using the RPPA technique to detect breast cancer-related proteins. Tumor tissue was lysed using a lysis buffer containing proteinase and phosphatase inhibitors. The lysates were diluted to a concentration of 1.33 mg/ml and boiled with SDS and 2-mercaptoethanol. The supernatants were further diluted in serial dilutions with lysis buffer. These samples were then spotted and immobilized on nitrocellulose-coated FAST slides. Probing with primary antibodies, the signal intensity was captured, amplified, and analyzed using MicroVigene software. The spot signal intensities were processed using SuperCurve (version 1.01) to derive protein concentrations. The concentrations were log2-transformed and normalized using median centering for each antibody across the samples. The samples were categorized into five groups (basal, luminal, HER2, and reactive I and II as defined in The Cancer Genome Atlas Network data set [16]) based on best-fitting consensus clustering.

### Metabolomics analysis

The concentration level of 18 metabolites was measured by high-resolution magic angle spinning magnetic resonance (HR-MAS MR) spectroscopy as previously described [21] using a Bruker Avance III 600 MHz/54 mm US (Bruker, Biospin GmbH, Germany) equipped with a 1H/13 C MAS probe. The mean sample weight was 7.3 ± 2.6 mg. Metabolites were quantified from Carr–Purcell–Meiboom–Gill (cpmg) pulse sequence (cpmgpr1d; Bruker) spin-echo spectra. The spectral region between 1.40 and 4.70 ppm was mean normalized after the removal of lipid residuals, and relative quantification was done by peak integration.

### Data analysis

#### Multi-omics factor analysis

Multi-omics Factor Analysis + (MOFA+) is an unsupervised data integration framework that allows for identification of the variation-explaining principal axes when multiple omics profiles of the same sample are analyzed concurrently [42]. These principal axes are low-dimensional interpretable representations of multi-omics datasets called latent factors (referred to as multi-omics factors, MOFs), thus allowing to perform downstream analyses such as clustering and feature selection.

MOFA+ training is carried out under a probabilistic Bayesian framework with an assumed prior joined distribution of unobserved variables within the model [42]. For $M$ different types of omics modalities (matrices) given as ${Y}_{1},\ldots {Y}_{m}$ with dimensions $N\times {D}_{m}$, where $N$ is the total number of samples and ${D}_{m}$ is the features available in the modality $m$, MOFA decomposes each ${Y}_{m}$ as (Eq1):

1 $${Y}_{m}=Z{W}^{{mT}}+{\epsilon }^{m}{;}\,m=1,\ldots ,M$$

Here, ${W}^{m}$ is the regularized weight matrix for each modality $m$, and ${\varepsilon }^{m}$ is the residual noise term specific to the modality. The latent factors matrix, Z, is a lower-dimensional representation of the original matrix Y, which stands common for all modalities. To determine the optimal MOFA+ model, four analogous MOFA+ models with latent factors of *n* = 10, 15, 20, and 30 dimensions were compared. The comparison was based on assessing the multicollinearity between the MOFs and the total variance explained (${R}^{2}$ by individual factors and modality) [43]. Average variational inflation factor (VIF) was used to analyze multicollinearity between the factors [43]. A high or low VIF is suggestive of a poor model. The spearman correlation coefficient was evaluated between the MOFs and the tumor’s characteristics. In addition, associations between clinical parameters (tumor grade, histology, tumor size, lymph node status, hormone receptor status) and MOFs were assessed by Wilcoxon and Kruskal–Wallis tests. For the association analyses, histology was dichotomized into two categories ductal and non-ductal type and tumor size was dichotomized into tumor size less than or larger than 20 mm. Further, a multivariate Cox proportional-hazards model was fitted over the MOFs to identify factors associated with patient prognosis. Schoenfeld test was used to test the proportionality of the hazards (PH) assumption of Cox model [44]. The significance of the hazard ratio and the overall significance of the multivariate models were evaluated using the likelihood ratio test (LRT) and Wald tests. For the Kaplan–Meier curve, a log-rank test was used to test the difference in the time-to-event curve between the different groups. Survival analysis was performed using the *“survival”* package. MOFA+ was implemented on *R Statistical Software (version 4.2.1)* using the “*MOFA2”* package [45].

#### Cluster analysis

Cluster analysis by k-means clustering was carried out on the survival-associated latent factors of the trained MOFA+ model. The Hartigan-Wong algorithm for K-means clustering was executed using Euclidean distance as the distance parameter [46]. Gap statistics and Silhouette scores were used to evaluate the optimal number of clusters, where the number of clusters (maximum 10) was chosen as the number corresponding to the largest GAP [47]. K-means clustering was implemented using the R (4.2.1) package *“stats”*, and the Gap statistics were obtained using the “cluster” package in R (4.2.1). (Supplementary Fig. 10).

### Validation framework

To test the robustness and clinical credibility of the found clusters on independent validation cohorts, a supervised learning-based validation scheme was used. We used two large-scale study cohorts for validation: The Cancer Genome Atlas (TCGA) and the Molecular Taxonomy of Breast Cancer International Consortium (METABRIC) (Table 1). From the validation cohorts, only those genes or proteins that were in common with the omics assays of the Oslo2 cohort were included in the validation scheme.

#### TCGA cohort

The Cancer Genome Atlas Pan-Cancer analysis project (TCGA-PanCanAtlas 2018) analyzed a large number of cancer tissues obtained from patients with primary tumors in different sites [48]. The study covered 12 tumor types including breast neoplasm. The data acquisition time for the TCGA began in 2006 (https://www.cancer.gov/ccg/research/genome-sequencing/tcga/history/timeline-milestones). Molecular profiles including the z-score normalized mRNA expression and the protein expression profiles for 1,084 primary BC samples along with their clinical information files were downloaded from cBioPortal (https://www.cbioportal.org/). To match the Oslo2 cohort, only patients with no history of neoadjuvant therapy were included in the analysis, resulting in 859 female BC samples.

#### METABRIC cohort

Molecular Taxonomy of Breast Cancer International Consortium (METABRIC) collected over 2000 primary BC tumor tissue samples from biobanks based in Canada and the UK [49]. The first study based on the METABRIC dataset was published in 2012 with a maximum follow-up of ~28 year [49]. This suggests that the compilation of the METABRIC dataset began around ~1985. These fresh-frozen samples are clinically annotated. The clinical information, gene expression data and metadata for 1904 samples were downloaded from cBioPortal (https://www.cbioportal.org/). Samples undergoing any therapy including chemotherapy or radiation therapy were excluded from the analysis. The final cohort comprised 694 samples.

Since the TCGA cohort comprises of transcriptome and protein profiles while the METABRIC cohort only offers transcriptomic profiles of BC patients, we perform a supervised analysis to overcome the incompleteness concerning the complete analysis performed on the Oslo2 cohort. As part of a sensitivity analysis, we also conducted validation across all samples in the cohort, regardless of their history of neoadjuvant therapy.

##### Supervised learning

Once the multi-omics clusters were identified in the Oslo2 cohort, multiple supervised classifiers were trained over multi-omics features set to classify the multi-omics clusters. These machine learning (ML) classifiers learned the intricate patterns embedded in the multi-omics assays in a supervised fashion which could be translated for the validation cohorts to predict the respective cluster of their samples. Three state-of-the-art machine learning classifiers were used in this study: random forest (RF), support vector machines (SVM) with linear kernel and partial least square discriminant analysis (PLS-DA) [50–52]. As an ensemble, the different mathematical foundations underlying these models offer a holistic approach to learning the intrinsic patterns, thus improving the reliability of the predictions (Supplementary Figs. 11–12) [53]. The RF, SVM and PLS-DA were implemented in R (4.2.1) using the packages *“randomForest”*, *“e1071”* and *“caret”* respectively.

To validate our results on the external cohorts, for each cohort, the supervised classifiers were trained over the Oslo2 cohort, by incorporating the set of common multi-omics features between Oslo2 and the external cohort (Fig. 1). Furthermore, to mitigate the challenge of dealing with high-dimensional data of the transcriptomic data, only the genes that exhibited differential expression (differentially expressed genes, DEGs) between the clusters in the Oslo2 dataset were utilized for the machine learning modeling. To identify DEGs, a significance analysis of microarrays (SAM) was performed for the Oslo2 mRNA profiles using the 21,851 genes with 100 permutations and a false discovery rate value of less than 0.01 (Supplementary Data 7). The *“siggenes”* package in R (4.2.1) was used to perform the SAM analysis.

The best-performing hyperparameters of the model were determined by assessing the model’s performance through 5-fold cross-validation and the area under the receiver operating characteristic (ROC) curve (AUC) [54] (Supplementary Data 5, 6) (Supplementary Figs. 11–12). The models were then trained for the entire Oslo2 cohort’s samples by setting the best-performing hyperparameters. Finally, these fully trained classifiers were fitted over the external cohort to predict the clusters for its samples. The cluster or class probabilities for each sample in the validation cohort were averaged across the trained classifiers and the class with the highest average probability was assigned to the sample.

### Integrated pathway analysis

To identify the enriched gene sets in the obtained MOCs, gene set enrichment analysis (GSEA) was implemented [55]. GSEA was performed pairwise on the multi-omics clusters. For the pairwise comparison, the ranking of the DEGs was based on the standardized size effect taking signal-to-noise ratio into account (Eq. 2) where ${\mu }_{N}$ is the mean and ${\sigma }_{N}$ is the standard deviation of the gene in cluster N, respectively (Supplementary Data 9).

2 $$\frac{{\mu }_{A}-{\mu }_{B}}{{\sigma }_{A}+{\sigma }_{B}}$$

KEGG database was referred to perform the GSEA [56]. Additionally, the gene sets C5 (gene ontology (GO) and Human Phenotype Ontology (HPO) gene sets) and C6 (oncogenic signature gene sets) derived from the Molecular Signatures Database (MSigDB) by The Broad Institute were also referred to evaluate the enrichments [57]. The gene sets database was filtered for the maximum and minimum size of 500 and 15 genes, respectively. For each comparison, *n* = 10,000 permutations were performed on phenotypes and the false discovery rate (FDR) cutoff was set to 25% (as recommended in the manual). The enrichment analysis was performed in R using the packages *“msigdbr”*, *“clusterProfiler”* and *“fgsea”*.

To further probe the obtained higher-order clusters at the molecular level, the top-weighted multi-omics features set was identified that contributed the most to the multi-omics latent factors. An integrated pathway analysis was carried out using the OmicsNet 2.0 software by incorporating these multi-omics features [58]. For network modeling, metabolite-protein was set as the primary network followed by protein-protein interaction as secondary molecular interaction referring to the STRING database [59]. A pairwise comparative study between the clusters was also performed. Pathways with an adjusted p-value less than 0.05 were considered significant. The network connections were identified using the current nodes and no new nodes were introduced.

### Supplementary information

Supplementary File Description

Supplementary Figure

Supplementary Data 1

Supplementary Data 2

Supplementary Data 3

Supplementary Data 4

Supplementary Data 5

Supplementary Data 6

Supplementary Data 7

Supplementary Data 8

Supplementary Data 9

## Data Availability

All code for data analysis associated with the current submission is available at https://github.com/AbhibhavS/BreastCancer-MultiOmics.

## Data availability

The supporting datasets of this article are included within the article Haukaas, T.H., et al., Metabolic clusters of breast cancer in relation to gene- and protein expression subtypes. Cancer Metab, 2016. 4: p. 12.; where mRNA expression data have been submitted to the Gene Expression Omnibus (GEO) database as a SuperSeries record with accession number GSE58215.
