# Supplementary material for: Comprehensive multi-omics analysis of breast cancer reveals distinct long-term prognostic subtypes
Source: Oncogenesis. 2024 Jun 13;13(1):22. doi: 10.1038/s41389-024-00521-6 (PMC11176181; doi:10.1038/s41389-024-00521-6)
Supplement: Supplementary file 1 — Supplementary File Description [file 41389_2024_521_MOESM1_ESM.docx]

**Supplementary File Description

Comprehensive multi-omics analysis of breast cancer reveals distinct long-term prognostic subtypes.**

**Abhibhav Sharma^1^, Julia Debik^1,2^, Bjørn Naume^3^, Hege Oma Ohnstad^4^, Oslo Breast Cancer Consortium (OSBREAC), Tone F. Bathen^2^, Guro F. Giskeødegård^1^**

1. Dept. of Public Health and Nursing (ISM), Norwegian University of Science and Technology- NTNU, Trondheim, Norway.
2. Dept. of Circulation and Medical Imaging, NTNU, Trondheim, Norway.
3. Department of Oncology, Division of Cancer Medicine, Oslo University Hospital, Oslo, Norway and Institute of Clinical Medicine, University of Oslo, Oslo, Norway
4. Department of Oncology, Division of Cancer Medicine, Oslo University Hospital, Oslo, Norway

**Supplementary Data 1**

Spearman’s Correlation coefficient between Factors and clinical features

(+ Pvalues of association test between Factors and clinical features)

**Supplementary Data 2**

Absolute loadings of 20 MOFs across Metabolites, Proteomes and Transcriptomes

**Supplementary Data 3**

Kruskal Wallis test between MOCs across the multi-omics

**Supplementary Data 4**

Common features between OSLO2 and external cohorts

**Supplementary Data 5**

Supervise ML 5-fold CV confusion matrix for TCGA cohort validation.

**Supplementary Data 6**

Supervise ML 5-fold CV confusion matrix for METABRIC cohort validation.

**Supplementary Data 7**

Pairwise SAM analysis results for MOCs

**Supplementary Data 8**

Multi-omics Network topology and the Multi-omics pathways through OmicsNet

**Supplementary Data 9**

Fold-change and P-value for the genes comparing MOCs pairwise

**Supplementary File:** *Supplementary Figures*
