# Supplementary material for: Comprehensive multi-omics analysis of breast cancer reveals distinct long-term prognostic subtypes
Source: Oncogenesis. 2024 Jun 13;13(1):22. doi: 10.1038/s41389-024-00521-6 (PMC11176181; doi:10.1038/s41389-024-00521-6)
Supplement: Supplementary file 2 — Supplementary Figure [file 41389_2024_521_MOESM2_ESM.docx]

**Supplementary Figure**

**Comprehensive multi-omics analysis of breast cancer reveals distinct long-term prognostic subtypes.**

**Abhibhav Sharma^1^, Julia Debik^1,2^, Bjørn Naume^3^, Hege Oma Ohnstad^4^, Oslo Breast Cancer Consortium (OSBREAC), Tone F. Bathen^2^, Guro F. Giskeødegård^1^**

1. Dept. of Public Health and Nursing (ISM), Norwegian University of Science and Technology- NTNU, Trondheim, Norway.
2. Dept. of Circulation and Medical Imaging, NTNU, Trondheim, Norway.
3. Department of Oncology, Division of Cancer Medicine, Oslo University Hospital, Oslo, Norway and Institute of Clinical Medicine, University of Oslo, Oslo, Norway
4. Department of Oncology, Division of Cancer Medicine, Oslo University Hospital, Oslo, Norway

***---------------------------------------------------------------------------------------------------------------------------***

***
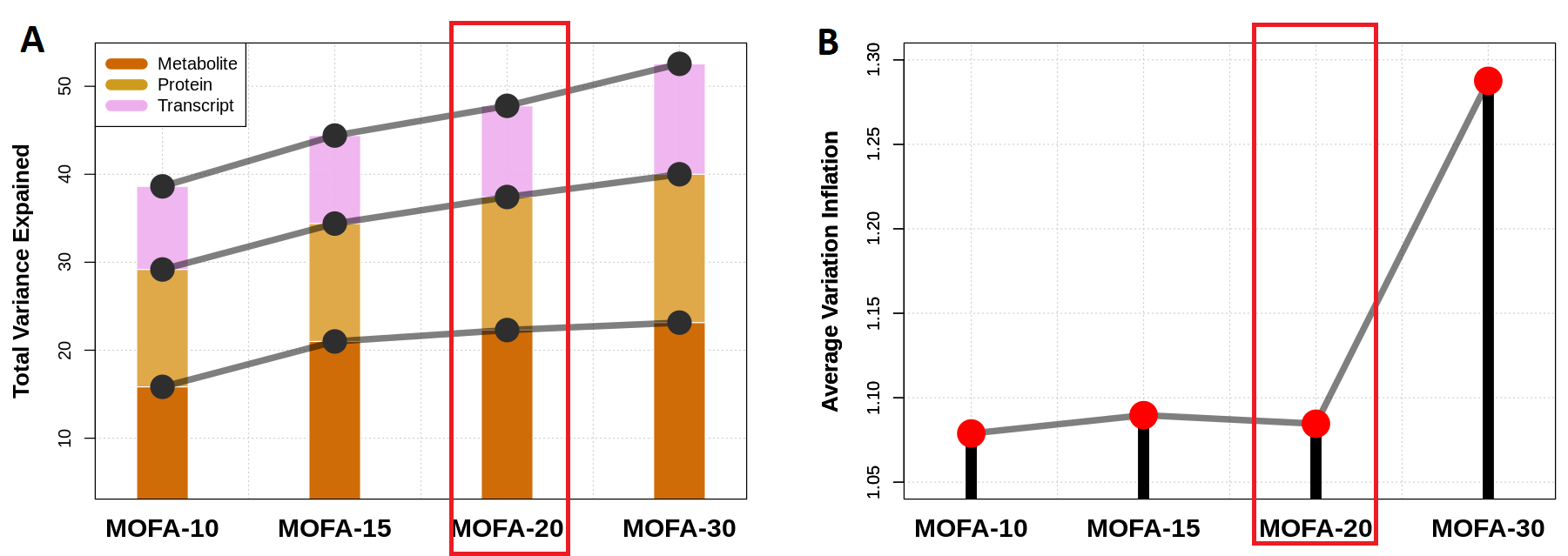
***

***Figure S1. a*** *Total variance explained by each modality for different latent factors MOFA+ model.* ***b*** *Average Variation inflation factor for the latent factor for MOFA+ models with varying degrees of latent factor.*

*
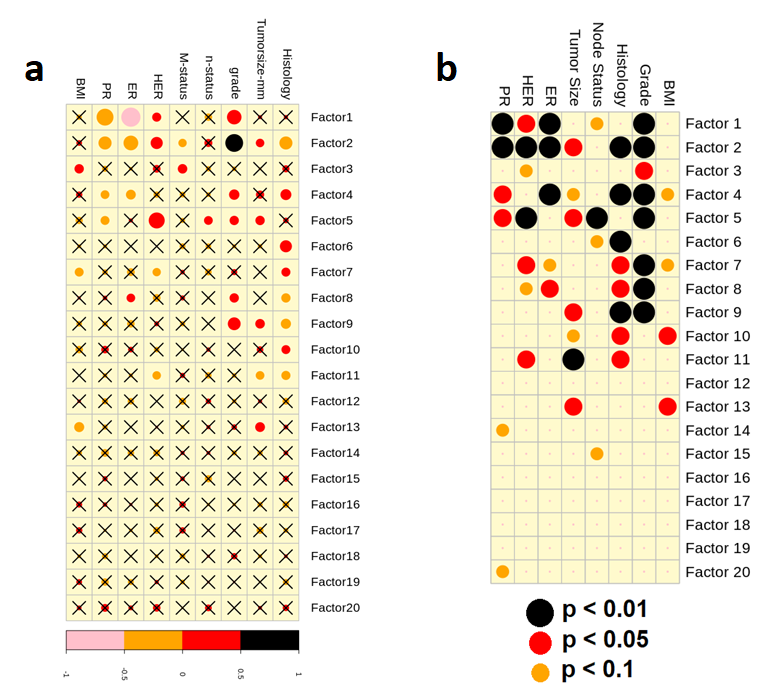
*

***Figure S2. a*** *Spearman’s Correlation between clinical features and MOFs. The cross indicates non-significant correlation (p < 0.05).* ***b*** *The p values for the test of association between the factors (continuous) and clinical features (categorical) are presented.*

*
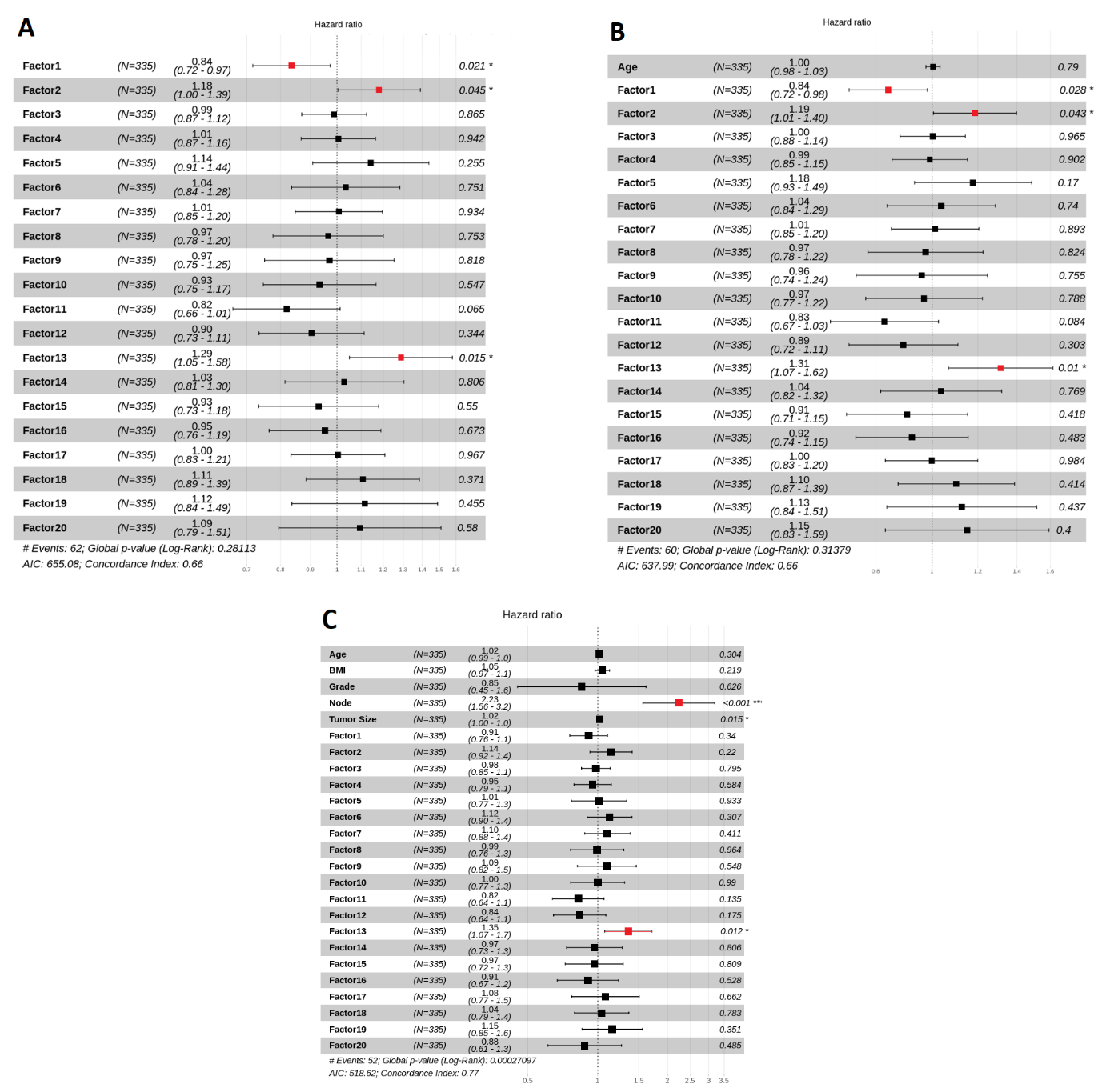
*

***Figure S3.*** *Forest plot for the multivariate Cox proportion hazard model for BC specific and metastatic event.* ***a*** *Without adjustment (Crude model),* ***b*** *With age adjustment c With age, BMI, tumor grade, tumor size and lymph node status adjusted.*

***
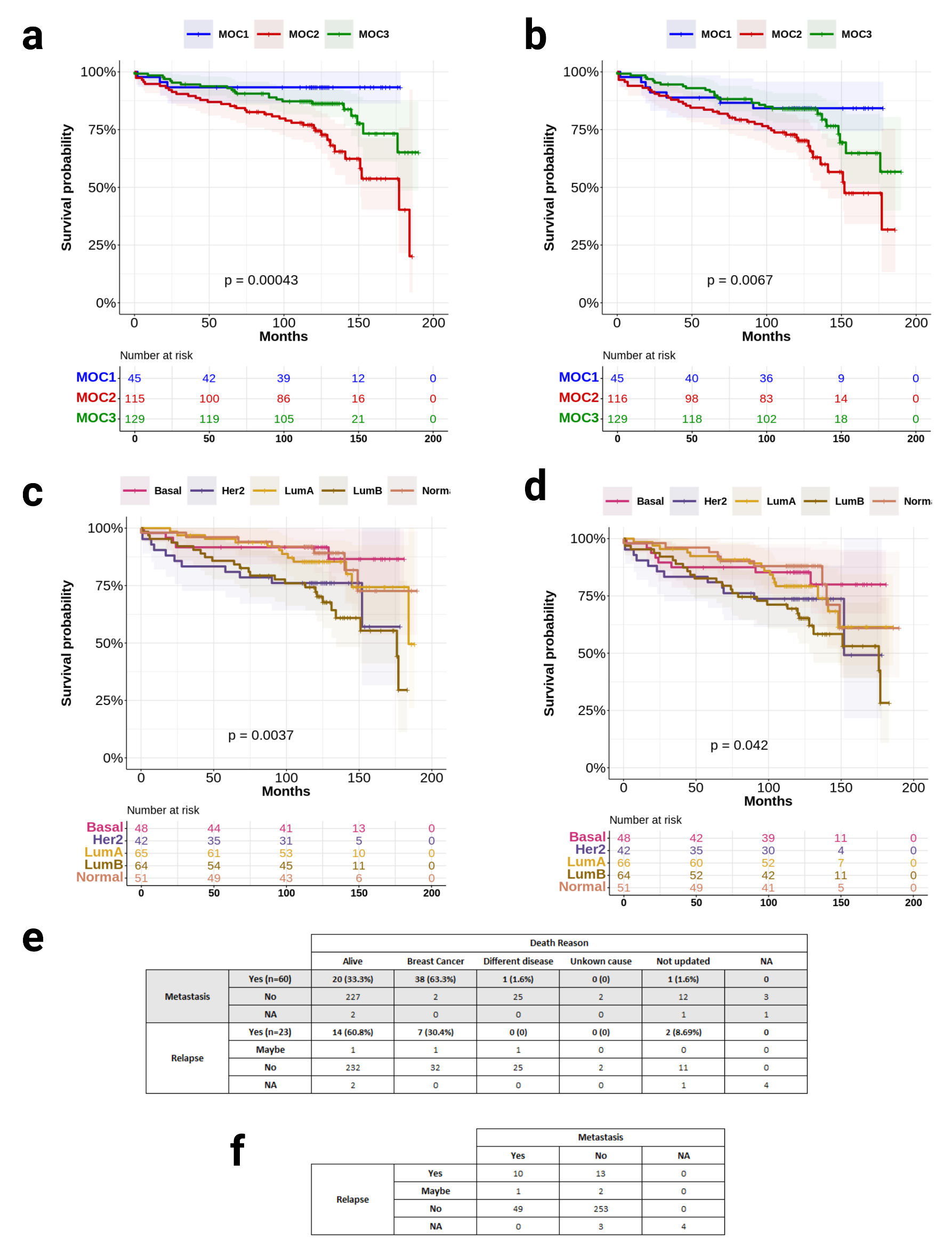
Figure S4.*** *Kaplan Meier survival plot for BC-specific death including metastasis in the Oslo2 cohort. for* ***a)*** *MOCs and* ***c)*** *Intrinsic subtypes****.*** *Kaplan Meier survival plot for BC-specific death including metastasis and relapse in the Oslo2 cohort. for* ***b*** *MOCs and* ***d*** *Intrinsic subtypes****.*** *Log-rank test P value is inscribed on the plot* ***e, f*** *Table showing the distribution of metastasis and relapse events in the Oslo2 cohort.*

*
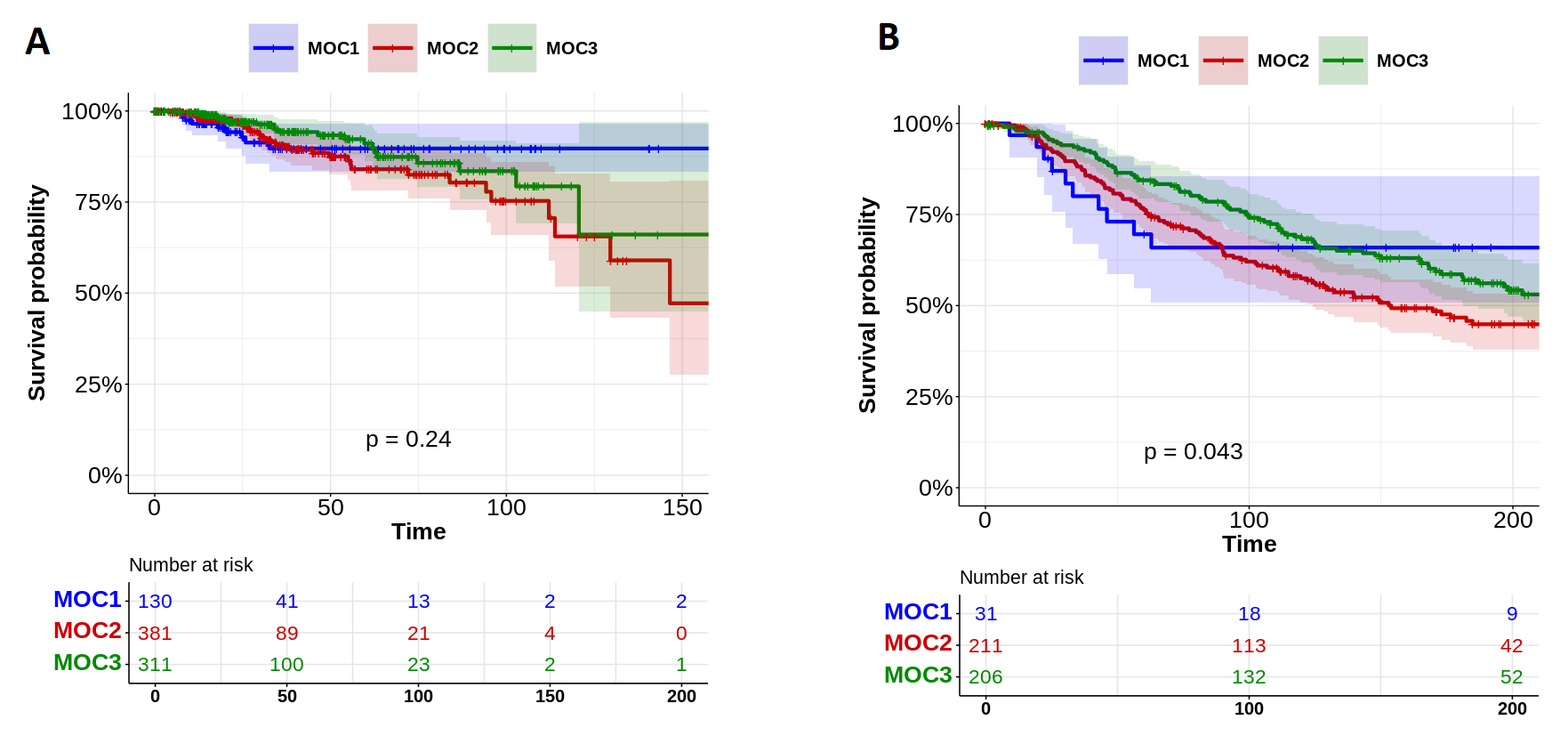
*

***Figure S5.*** *Kaplan Meier survival plot for only deaths due to breast cancer-* ***a*** *TCGA cohort.* ***b*** *METABRIC cohort. Log-rank test P value is inscribed on the plot.*

***
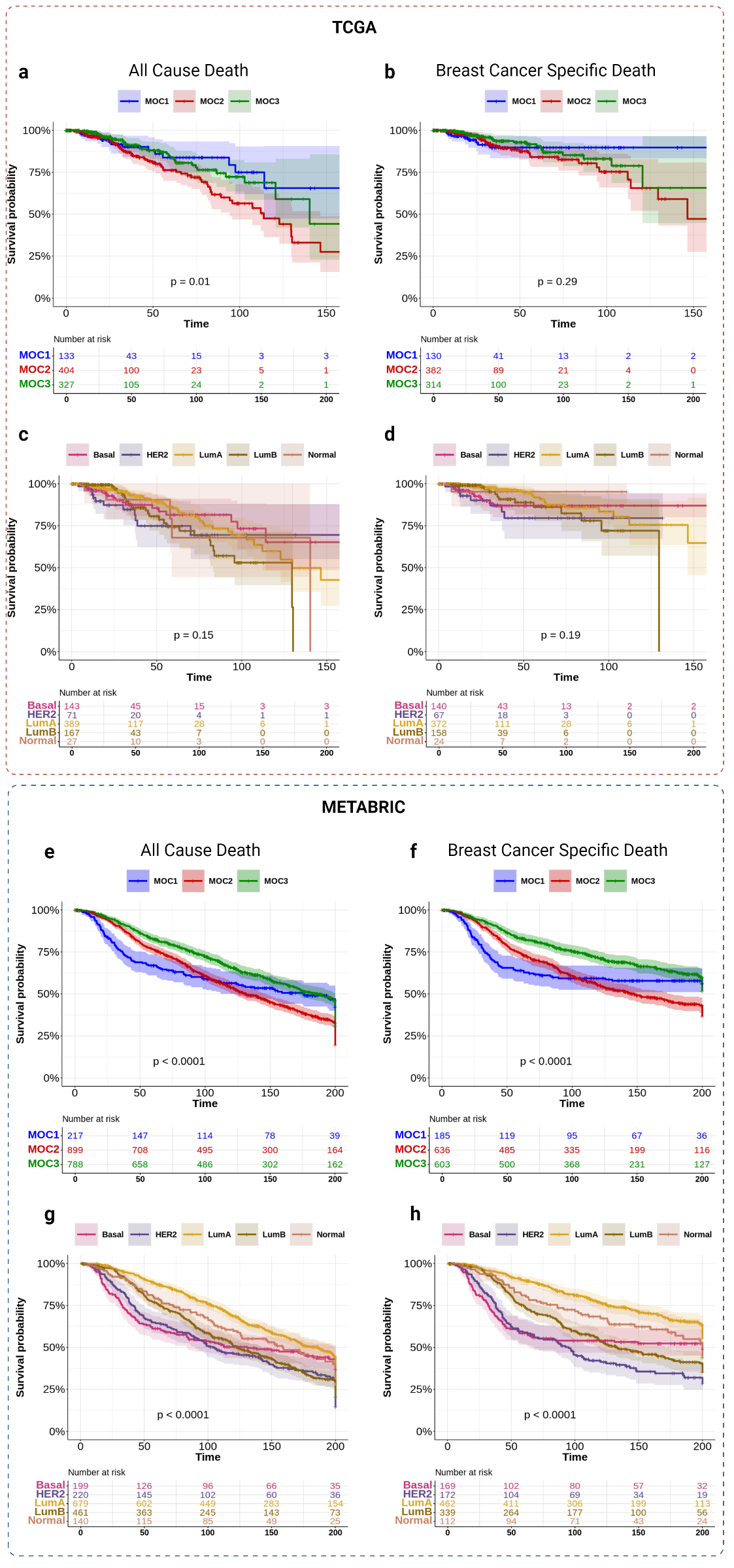
***

***Figure S6. Validation of the multi-omics clusters in external cohorts independent of neoadjuvant therapy status.*** *The Kaplan-Meier curves illustrate the overall long term-survival of MOCs and intrinsic subtypes in the TCGA (top) and the METABRIC cohort (bottom). The MOC’s survival curves for a), e) all cause death and b), f) breast cancer specific deaths in TCGA and METABRIC cohorts respectively. The intrinsic subtype’s survival curves for c), g) all cause death and d), h) breast cancer specific deaths in TCGA and METABRIC cohorts respectively. p-values from log rank test are shown.*

*
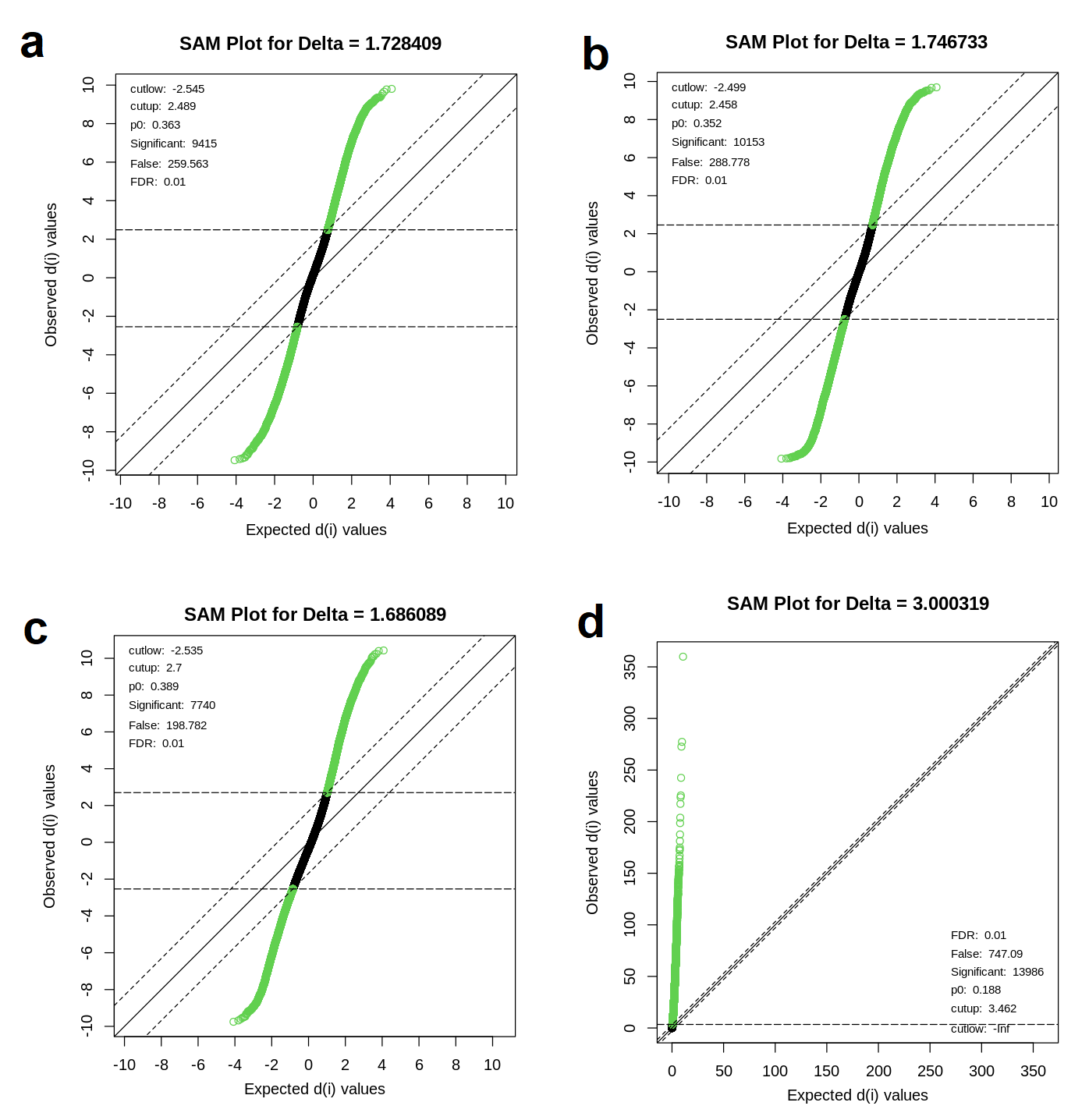
****Figure S7. SAM (Significance Analysis of Microarray) plots. a*** *SAM analysis between MOC1 and MOC2.* ***b*** *SAM analysis between MOC1 and MOC3****. c*** *SAM analysis between MOC2 and MOC3.* ***d*** *SAM analysis between MOC1 and MOC2 and MOC3. Green dots represent the DEGs.*

*
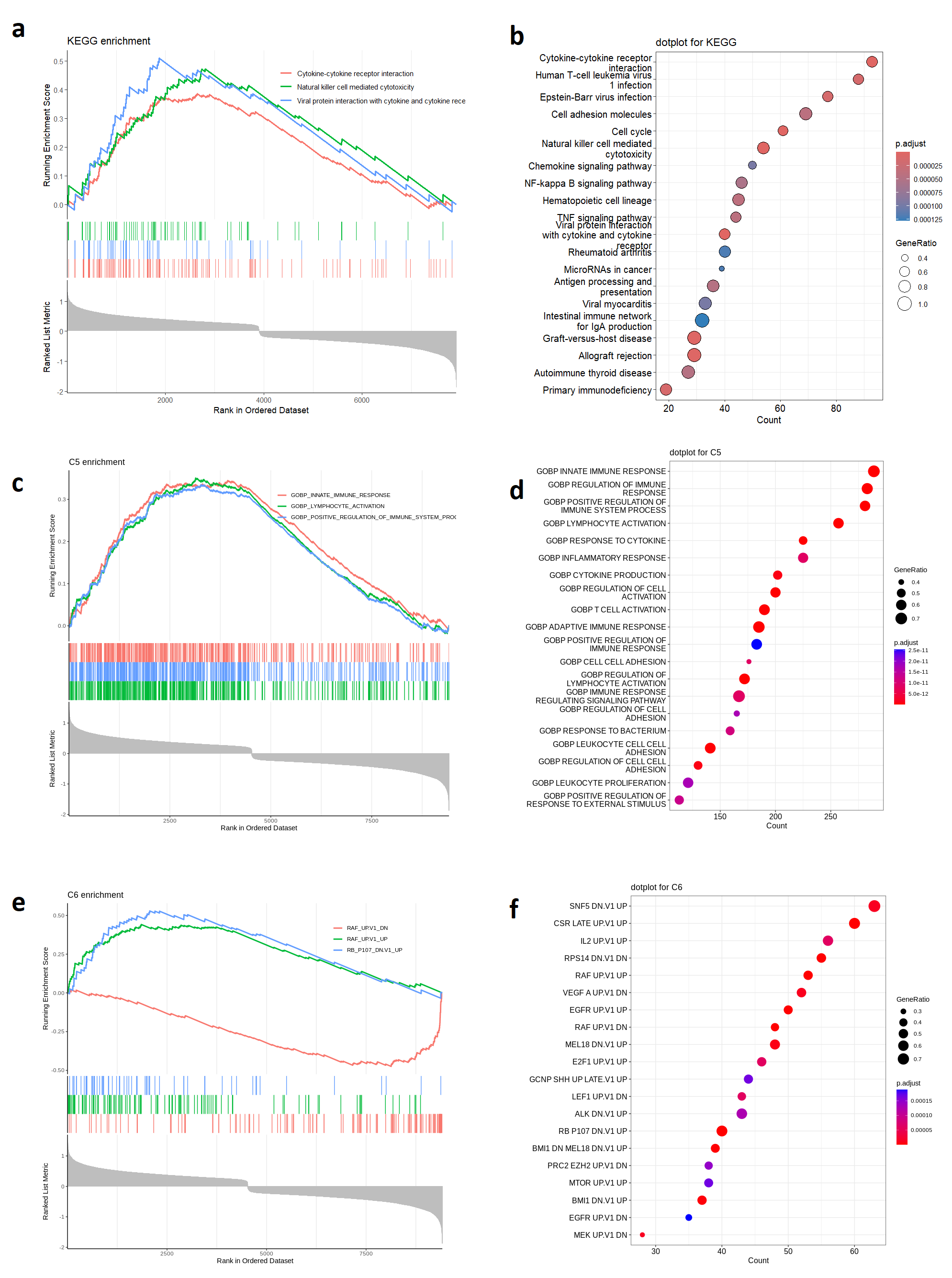
****Figure S8. GSEA analysis. a c d*** *GSEA enrichment plot for MOC1 vs MOC2 by referring to KEGG, C5 and C6 databases.* ***b d f*** *Dotplot for the GSEA analysis referring to KEGG, C5 and C6 database.*

*
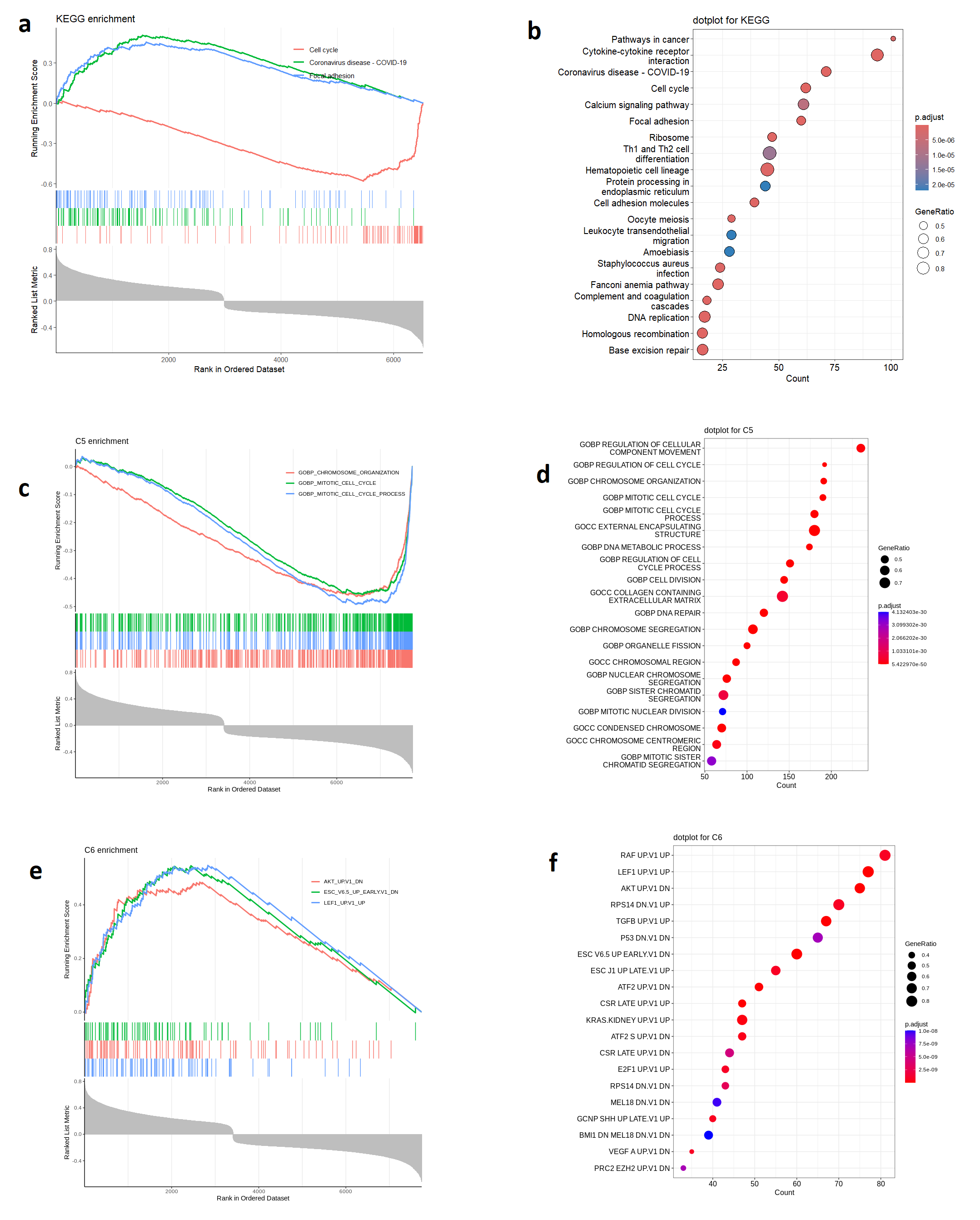
*

***Figure S9. GSEA analysis. a c d*** *GSEA enrichment plot for MOC3 vs MOC2 by referring to KEGG, C5 and C6 databases.* ***b d f*** *Dotplot for the GSEA analysis referring to KEGG, C5 and C6 database.*

***
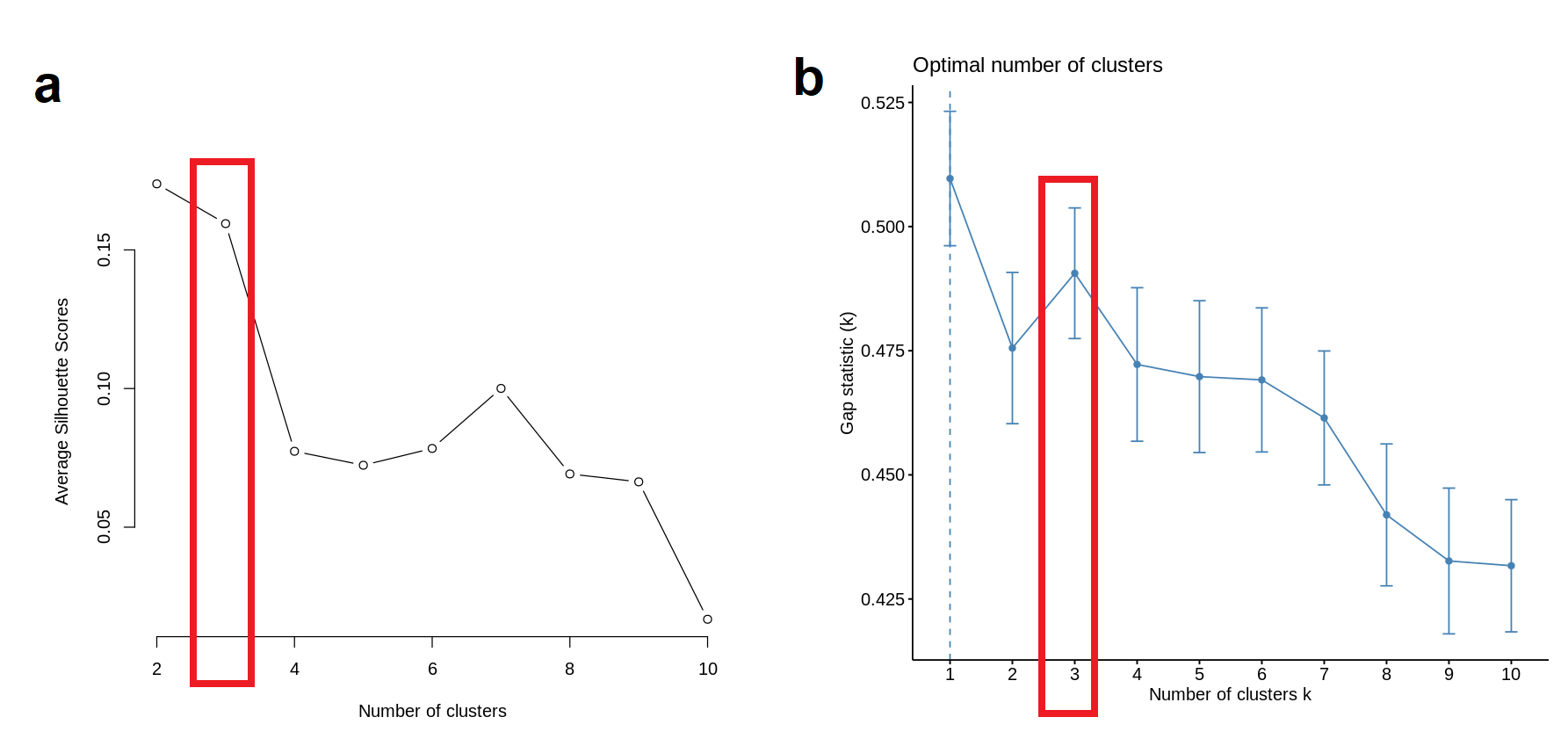
***

***Figure S10. a*** *Average Silhouette score obtained for different choices of k (number of clusters).* ***b*** *GAP statistic obtained for different choices of k (number of clusters). The red box indicates the elbow point translating into the desired number of k.*

*
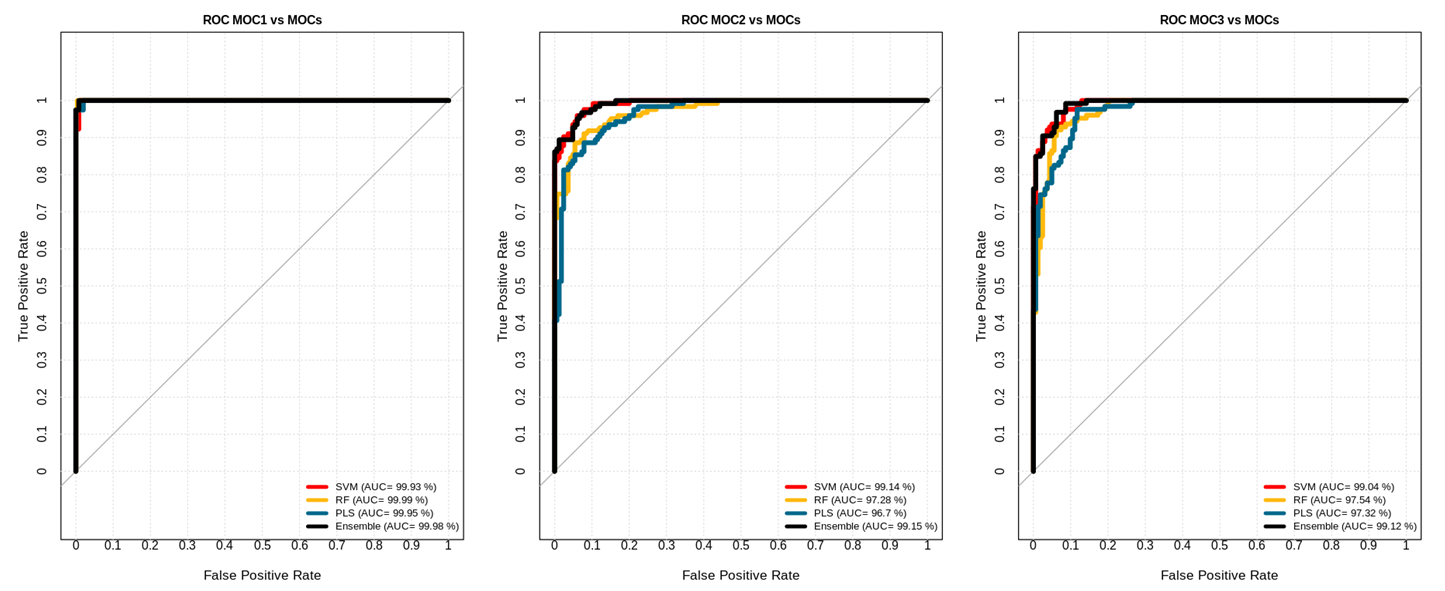
*

***Figure S11.*** *ROC curve for supervised ML model and ensemble model (in black) obtained during 5 fold CV of Oslo2 data by incorporating the features common between TCGA and Oslo2 cohort.*

*
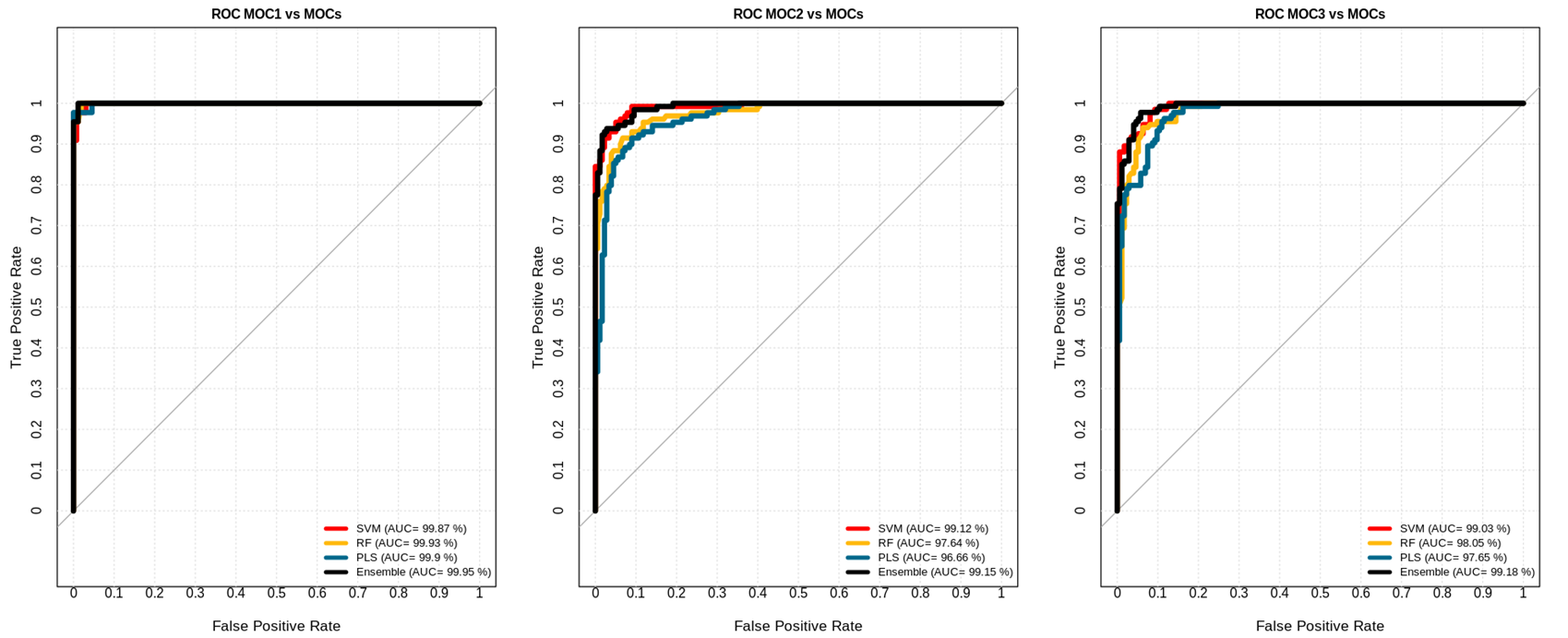
*

***Figure S12.*** *ROC curve for supervised ML model and ensemble model (in black) obtained during 5 fold CV of Oslo2 data by incorporating the features common between METABRIC and Oslo2 cohort.*

R packages used:

“survival” [1]

“MOFA2” [2]

“stats” [3]

“cluster” [4]

“randomForest” [5]

“e1071” [6]

“caret” [7]

“siggenes” [8]

“msigdbr” [9]

“clusterProfiler” [10]

“fgsea” [11]

1. Therneau, T.M., A Package for Survival Analysis in R. 2022.
2. Argelaguet, R., et al., MOFA+: a statistical framework for comprehensive integration of multi-modal single-cell data. Genome Biol, 2020. 21(1): p. 111.
3. Team, R.C., R: A Language and Environment for Statistical Computing. 2022.
4. Maechler, M., et al., cluster: Cluster Analysis Basics and Extensions. 2022
5. Liaw, A. and M. Wiener, Classification and Regression by randomForest. R News, 2002. 2(3): p. 18-22.
6. Meyer, D., et al., e1071: Misc Functions of the Department of Statistics, Probability Theory Group (Formerly: E1071), TU Wien. 2022.
7. Kuhn, M., caret: Classification and Regression Training. 2022.
8. Schwender, H., siggenes: Multiple Testing using SAM and Efron's Empirical Bayes Approaches. 2022.
9. Dolgalev, I., msigdbr: MSigDB Gene Sets for Multiple Organisms in a Tidy Data Format. 2022.
10. Wu, T., et al., clusterProfiler 4.0: A universal enrichment tool for interpreting omics data. Innovation (Camb), 2021. 2(3): p. 100141.
11. Korotkevich, G., V. Sukhov, and A. Sergushichev, Fast gene set enrichment analysis. bioRxiv, 2019.

All codes are available at:
[AbhibhavS/BreastCancer_MOFA (github.com)](https://github.com/AbhibhavS/BreastCancer_MOFA)
